# Does invasive surgical acceleration of orthodontic tooth movement increase root resorption? A harms-focused systematic review

**DOI:** 10.1371/journal.pone.0356175

**Published:** 2026-08-19

**Authors:** Mohamad Radwan Sirri, Mohammad Osama Namera, Mohamad Yaman Salahi Alasbahi, Zaher Alswaidan, Salar Karim Khalil

**Affiliations:** 1 Department of Orthodontics, Faculty of Dentistry, Damascus University, Damascus, Syria; 2 Department of Restorative Dentistry and Endodontics, Faculty of Dentistry, Damascus University, Damascus, Syria; 3 Department of Medical Laboratory Technology, Technical Institute of Zakho, Duhok Polytechnic University, Duhok, Iraq; International Medical University, MALAYSIA

## Abstract

**Background:**

Orthodontically induced root resorption (RR) is frequent, and invasive surgically accelerated orthodontic (ISAO) procedures may increase it. This study aimed to assess the effect of ISAO on RR in teeth adjacent to surgically treated segments compared with conventional orthodontic treatment.

**Methods:**

Following PRISMA, five major databases and four grey literature sources were searched through August 2025 without language or date restrictions. Eligible studies were randomized controlled trials and comparative non-randomized intervention studies, including split-mouth designs, that reported quantitative root resorption after flap-based procedures. Two reviewers independently selected studies, extracted data, and assessed risk of bias using RoB 2 and ROBINS-I. Root resorption ascertainment and harms reporting were appraised with a modified McMaster Harms tool, and certainty was evaluated with GRADE. The review was registered in PROSPERO (CRD420251166274).

**Results:**

Eleven studies involving 352 patients were included. Because of substantial heterogeneity in surgical protocols, imaging methods, root resorption metrics, and follow-up periods, findings were synthesized narratively. In cone-beam computed tomography studies, plain corticotomy showed either small increases or no clear between-group differences in root resorption. Some graft-augmented corticotomy or osteotomy protocols were associated with slightly lower measured root resorption than conventional controls. In two-dimensional or index-based assessments, most comparisons showed no clear difference. Across studies, observed root resorption generally remained within commonly accepted clinical limits, but evidence certainty was low to very low.

**Conclusions:**

Current low- to very low-certainty evidence does not demonstrate a large increase in root resorption with invasive surgically accelerated orthodontics. However, the available studies are small, heterogeneous, and frequently at high risk of bias, preventing firm conclusions about safety. Findings suggesting slightly lower measured root resorption with some graft-augmented protocols remain hypothesis-generating and require confirmation in larger, better-designed studies with standardized outcomes and harms reporting.

## Introduction

Orthodontically induced root resorption (RR) is defined as an irreversible loss of root structure at the apex resulting from an inflammatory response within the periodontal ligament and alveolar bone under applied orthodontic forces [1]. Biologically, this process begins when orthodontic loading compresses the periodontal ligament microvasculature and disturbs local cellular homeostasis, leading to sterile inflammation, focal hyalinization, and recruitment of clastic cells capable of resorbing cementum and, in more pronounced cases, the underlying dentin. Local cytokines and osteoclastogenic signaling mediate this response, and its extent appears to reflect the balance between tissue injury and reparative capacity [2]. Mild to moderate RR is common, occurring in approximately 40–60% of patients, whereas severe RR remains an unpredictable minority [3]. Recent reviews estimate severe cases at about 1–5%, although estimates vary with the definition of severity and diagnostic thresholds [3]. Rather than being determined solely by the application of force, RR is now regarded as a multifactorial phenomenon. Susceptibility may be modified by patient-related factors, such as individual biological or genetic predisposition and previous dental trauma; tooth-related factors, such as the treated tooth, root morphology, and initial root form; and treatment-related factors, such as force magnitude and continuity, treatment duration, extraction mechanics, and movements that concentrate stress at the apex, particularly intrusion and torque. Recent CBCT-based evidence has also suggested that some demographic and malocclusion-related variables may contribute to RR expression. However, these associations are not fully consistent across tooth types and study designs [1,4]. With two-dimensional imaging, thresholds of 2–4 mm of radiographically detectable root shortening are typically considered clinically relevant. In contrast, CBCT allows earlier three-dimensional detection, provided that its use is appropriately justified [5,6].

To shorten orthodontic treatment duration, invasive surgically accelerated orthodontics (ISAO) techniques have been proposed to exploit the regional acceleratory phenomenon (RAP), a transient, surgery-induced increase in bone turnover and remodeling. Flap-based ISAO is commonly categorized into three groups.

First, corticotomy-based methods involve creating shallow cortical cuts to trigger the RAP. These include selective alveolar corticotomy, interradicular corticotomy, and periodontally accelerated osteogenic orthodontics (PAOO), in which particulate bone grafts are added to augment the alveolus [7]. Second, segmental or block osteotomy uses full-thickness osteotomies around a dentoalveolar segment to allow en-masse repositioning when orthodontic mechanics alone are insufficient [8]. Finally, distraction techniques include dentoalveolar distraction for rapid canine retraction using a distractor and alveolar distraction osteogenesis for gradual segment movement [9].

These approaches differ in surgical extent (superficial decortication vs full osteotomy), instrumentation (rotary/microsaw/piezosurgery), biomaterial use (mandatory grafting in PAOO), and the scale/rate of movement (tooth-focused acceleration vs block movement) [10]. The literature often mixes these invasive techniques with minimally invasive surgically accelerated orthodontics (MISAO), such as flapless piezocision or micro-osteoperforations, so clear separation is essential when assessing adverse effects [7,11,12].

Initial clinical trials on ISAO have reported conflicting findings regarding RR. Some studies suggest that accelerated mechanics may increase the risk of root shortening [13], while others report no difference [14] or even slightly reduced RR [15] compared with conventional therapy. These discrepancies warrant critical appraisal of the evidence, with attention to differences in surgical technique, imaging modality (CBCT vs periapical radiographs), and applied biomechanics.

Across contemporary reviews, the relationship between ISAO and RR has often been treated as a secondary or safety outcome rather than a primary focus [16–18]. As a result, RR outcomes have been inconsistently defined, variably measured, and frequently underreported. In addition, many reviews have pooled invasive and minimally invasive procedures together under broad labels such as “surgically accelerated orthodontic treatment,” further obscuring potential differences in harms between techniques [7,19].

A harm-focused, methodologically robust evaluation of RR in ISAO is therefore required. The present review adopts a structured harms-first approach using the modified McMaster Harms (McHarm) tool to assess the quality of RR outcome measurement and reporting [20]. This framework is consistent with the CONSORT-Harms 2022 extension for randomized trials [21] and the PRISMA-Harms guidance for systematic reviews [22], and it aligns with Cochrane Handbook recommendations that adverse effects require tailored methods and dedicated reporting [23].

Accordingly, this harm-focused systematic review aims to estimate the effect of ISAO on RR in teeth adjacent to surgically treated segments, compared with conventional orthodontic treatment, and to critically appraise the certainty of the available evidence.

## Materials and methods

### Scoping search and protocol

A preliminary scoping search of PubMed was conducted to determine whether harm-focused systematic reviews specifically addressing RR in ISAO were already available, and none were identified. The review protocol was then developed in accordance with the PRISMA harms checklist to optimize adverse-event reporting [22] and the Cochrane Handbook for Systematic Reviews of Interventions (version 6.5) [23]. The protocol was prospectively registered in PROSPERO (CRD420251166274).

### Eligibility criteria

Inclusion criteria were defined a priori using the PICOS framework (Population, Intervention, Comparison, Outcome, Study design; Table 1).

**Table 1:** PICOS framework and the searched electronic databases.

Participants	Healthy individuals of any age, sex, or ethnicity receiving active orthodontic treatment (fixed appliances or clear aligners), with or without extraction plans; no restrictions on malocclusion type or treatment indication.
Interventions	Any orthodontic treatment (with or without extraction) assisted by invasive surgical techniques to accelerate orthodontic movement (e.g., corticotomy, periodontally accelerated osteogenic orthodontics, osteotomy, distraction) was included.
Comparisons	A matched control group with appliances but without additional intervention to accelerate tooth movement.
Outcomes	RR quantitatively measured by conventional radiographs (i.e., IOPA and panoramic radiographs) or tomographic imaging (i.e., μCT, CT, and CBCT).
Study design	Randomized controlled trials and comparative non-randomized studies of interventions with a concurrent or matched control group were eligible. Split-mouth designs were included.

RR: root resorption, IOPA: intraoral Periapical; μCT: micro-computed tomography; CT: computerized tomography, CBCT: cone beam computed tomography

#### Exclusions.

(i) animal studies; (ii) studies with fewer than five participants per group to reduce the influence of small and unstable samples; (iii) non-surgical interventions or minimally invasive surgically accelerated orthodontic (MISAO) techniques without flap (e.g., piezocision, micro-osteoperforations); (iv) duplicate publications, overlapping samples, or preliminary reports superseded by more complete data; and (v) studies without extractable quantitative RR outcomes or without a clear description of measurement methods.

### Search strategy

Two reviewers (MRS and MON) independently conducted a comprehensive electronic search from database inception through August 2025, with no restrictions on language, publication year, publication status, malocclusion type, or treatment indication. Sources included PubMed, Web of Science, Scopus, Embase, and the Cochrane Central Register of Controlled Trials (CENTRAL), supplemented by Google Scholar and Trip; grey literature was queried via OpenGrey and ProQuest PQDT Open (dissertations and theses). Full database-specific strategies were provided in S1 Table.

### Study selection and data extraction

Titles and abstracts were screened independently by **two reviewers** (ZA, MON) against prespecified eligibility criteria, and any disagreements were resolved by a third reviewer (MYSA). Potentially eligible records underwent full-text assessment, with reasons for exclusion documented.

Data were extracted using a standardized, pilot-tested form capturing: study setting (author, country, year, design); participant characteristics (sample size, sex, age range/mean); methods (treatment comparisons, appliance system, archwire sequence, force magnitude/device, extractions [yes/no], anchorage type); intervention details (type and site, dimensions and technique of the surgical procedure, graft use, device used); follow-up and duration (repeat interventions, follow-up schedule, total treatment time); and root resorption assessment and outcomes (measurement methods and statistical significance between experimental and control groups).

### Risk of bias and quality of RR assessment

Risk of bias was assessed with the Cochrane RoB 2 tool for randomized clinical trials [24] and ROBINS-I for non-randomized controlled clinical studies [25], following the developers’ guidance.

To appraise the quality of root-resorption (RR) ascertainment across both designs, a modified McMaster Harms (McHarm) tool was used, as it captures harm-specific biases with item-level criteria and complements standard risk-of-bias tools [20]. Operationally, an abridged 11-item McHarm set was rated as Yes/No/Unclear for every included study (randomized and non-randomized). Items covered whether RR was prospectively specified and precisely defined; identification of key risk modifiers (e.g., force magnitude/duration, tooth type); who collected and adjudicated RR measurements; timing and frequency of assessments; use of standardized scoring or imaging protocols; handling of withdrawals and missing data; and transparency of statistical methods for harm analyses. A “Yes” rating indicated lower concern for harm-specific bias (greater clarity, completeness, and internal validity of RR reporting), whereas “No” signaled important concerns, and “Unclear” indicated insufficient reporting. This McHarm-based appraisal complements RoB 2/ROBINS-I by targeting domains unique to adverse-event capture and aligns with contemporary CONSORT-Harms 2022 guidance on prespecification, denominators/time-at-risk, and transparent harms reporting [26].

Two independent reviewers (MRS, MON) performed duplicate assessments; disagreements were resolved by a third reviewer (MYSA). Domain-level judgments for RoB 2, ROBINS-I, and McHarm are summarized in S2 Table.

### Certainty of evidence (ISAO and RR)

Certainty of evidence for each treatment type was appraised using the Grading of Recommendations Assessment, Development, and Evaluation approach (GRADE) [27], with ratings categorized as high, moderate, low, or very low. Assessments were performed independently by two authors (MRS, MON); any discrepancies were resolved by a third author (MYSA).

### Summary measures and synthesis

Given the considerable clinical and methodological heterogeneity across the included studies, a quantitative meta-analysis was not performed. Heterogeneity arose from differences in surgical protocols (type, extent, and adjunctive grafting), imaging modalities (CBCT, periapical radiography, or mixed two-dimensional approaches), RR outcome metrics (root length, tooth length, or categorical indices), and follow-up intervals. Instead, the results were synthesized narratively using a structured approach. Studies were grouped first by treatment objective and then interpreted within strata defined by imaging modality and RR metric. Absolute numerical changes in millimeters were not compared across different imaging modalities or against categorical indices, and follow-up duration was treated as an important interpretive context rather than as a directly comparable dimension. Accordingly, numerical differences were considered comparable only within broadly similar measurement frameworks and clinical contexts.

Where relevant, randomized and non-randomized evidence were synthesized and interpreted separately. Greater interpretive weight was given to randomized evidence, whereas non-randomized findings were considered complementary and hypothesis-generating because of their greater susceptibility to confounding.

## Results

### Search flow and study selection

**Fig 1:**
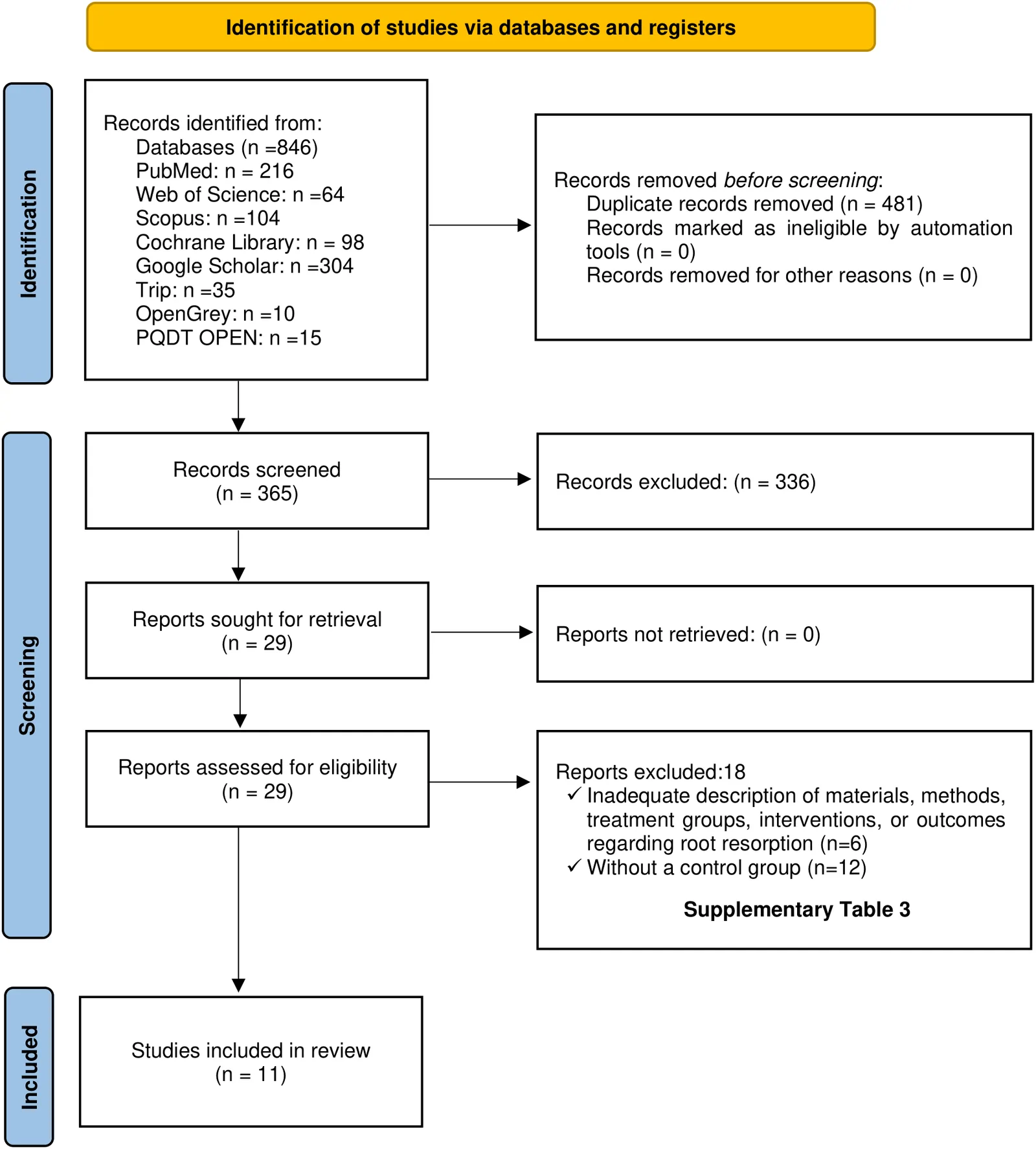
Preferred Reporting Items for Systematic Reviews and Meta-Analyses (PRISMA) flow diagram of the included studies. Source: Created by the authors.

The comprehensive search identified 846 studies. After deduplication, 365 studies proceeded to title and abstract screening. Application of the prespecified eligibility criteria led to the exclusion of 336 records. The full texts of the remaining 29 articles were assessed in detail; 18 were excluded, with reasons listed in S3 Table. Accordingly, 11 studies were included in the present systematic review. The study selection pathway is depicted in the PRISMA flow diagram (Fig 1).

### Characteristics of the included studies

The review included 11 studies conducted between 2013 and 2025, enrolling 352 participants (approximately 1329 teeth), with sample sizes ranging from 10 to 56 and ages from 14 to 40 years, and most cohorts comprising both genders (Table 2). Study designs comprised six RCTs, two with a parallel design [13,28] and four with a split-mouth design [29–32], and five non-randomized studies, four parallel [14,15,33,34] and one split-mouth [35]. Treatment objectives covered leveling and alignment in two trials [13,15], canine retraction in four trials [29–32], en-masse retraction in two trials [28,33], and presurgical decompensation in three trials [14,34,35]. All comparisons evaluated ISAO procedures against conventional orthodontic treatment. Interventions included four corticotomy protocols [13,29,30,32], four corticotomy with graft protocols [14,31,34,35], two PAOO with graft protocols [15,28], and one anterior segmental osteotomy [33]. A single intervention session was performed in every study, with labial approaches in eight studies and combined labial and lingual approaches in three. Evaluation periods varied by objective, spanning 6–7 months for alignment and leveling, 3–7 months for canine retraction, 12–28 months for en-masse retraction, and 8–23 months for presurgical decompensation. Diagnostic methods included CBCT in seven studies [13,14,29,31,33–35] and periapical radiography in four [15,28,30,32]. Root resorption was assessed using tooth length [15,31,33], root length [14,34,35], the Malmgren index [32], or the Sharpe index [28]. In three studies, the RR assessment method was not clearly specified [13,29,30]. Characteristics of the included studies are summarized in Tables 2-3.

**Table 2:** Characteristics of included studies in the systematic review.

Treatment goal	Study setting	Participants		Methods						Intervention					Follow-up & Duration		
	Author / Country / Year Study design	Sample size (F/M)	Age (years)	Treatment comparison	Appliance system	Archwire sequence	Force magnitude & device	Time of extraction	Anchorage type	Type & site of intervention	Dimensions of surgical intervention	Surgical technique	Graft use	Device used	Repeat intervention	Follow-up	Total duration
**Alignment & leveling**	**Alam et al** 2025 Saudi Arabia Parallel RCT	50 (NR)	18–30 (mean NR)	**2 arms:** ACAO vs Conventional	0.022″ edgewise	NiTi (NR)	Light continuous NiTi	Not reported	Not reported	**Labial + Lingual** (full flap corticotomy)	Not specified	Vertical + horizontal piezo cuts	No	Piezosurgery	Single	Until debonding	ACAO: 10.5 ± 1.7 mo vs Ctrl: 16.3 ± 2.2 mo
	**Ferguson et al** 2016 UAE + USA Parallel Retrospective	54 (27 + 27)	PAOO: 24.8 ± 10.2; Conv: 19.6 ± 8.8	**2 arms:** PAOO vs Conventional	Edgewise 0.022	Progressive	NR	Non-extraction	Conventional	**Labial + Lingual** (anterior maxilla)	Not specified	Decortication + augmentation	Yes	Bur + graft	Single	Until debonding	PAOO: 6.3 ± 2.4 mo vs Ctrl: 17.2 ± 6.5 mo
**Canine retraction**	**Abbas et al** 2016 Egypt SMD RCT	20 (NR)	15–25	2 arms Group 1: Corticotomy (test) vs contralateral control; Group 2: Piezocision (test) vs contralateral control	Roth 0.022 (straight-wire)	0.016 × 0.022 stainless steel engaged immediately post-op after leveling/alignment	150 g closed-coil NiTi per side; checked biweekly with a tension gauge	One first premolar, the day before surgery; contralateral on surgery day	Conventional anchorage: tie the upper first molar with the second premolar; retract from the canine hook to the molar band hook	Labial (buccal) cortical plate only around the maxillary canine	Corticotomy: submarginal flap 4 mm; vertical corticotomies 2–3 mm apical to crest along root + facial cortical perforations; remove bundle bone from mesial socket wall.	Corticotomy with full-thickness flap and suturing; Piezocision flapless (micro-incisions) with suturing	No	Piezotome (NSK VarioSurg3); Dentaurum tension gauge	Single	3 months with biweekly measurements	3 months assessment after alignment
	**Baiomy et al** 2021 Egypt SMD RCT	24 (16F/8M)	14–22 (mean 18)	**3 arms:** Group I Vertical corticotomy vs Group II Modified (vertical + perforations) vs Control	0.022″ fixed	To 0.018 SS	150 g NiTi coil	After leveling, before corticotomy	TADs (1.6 × 9 mm)	**Labial only** (maxillary canines)	Not specified (vertical grooves along root length)	Vertical vs vertical + perforations	No	Round bur	Single	6 mo	G I: 136.9 d vs Ctrl 152 d; G II: 120.4 d vs Ctrl 151 d
	**Raza et al** 2021 India SMD RCT	10 (NR)	15–25	**2 arms:** Corticotomy (vertical + perforations) vs Control	MBT 0.022 (3M)	To 0.018 SS	150 g NiTi coil	After leveling, before corticotomy	TPA + Nance	**Labial only** (maxillary canines)	Not specified (vertical + perforations labially)	Vertical + perforations + graft	Yes	Bur + graft	Single	7 mo	Test: 5.7 mo vs Ctrl: 7.1 mo
	**Sharma et al** 2020 India SMD RCT	14 (NR)	14–25 (mean 18.9)	**2 arms:** Corticotomy distal to canine vs Control	MBT 0.022	To 0.016 × 0.022 SS	150 g NiTi coil	Day of corticotomy	TPA + molars banded	**Labial only** (distal to canine)	Horizontal cut 2 mm apical; vertical groove distal to the canine; perforations in the socket	Vertical + horizontal grooves, bur	No	Round bur	Single	6–7 mo	Test faster first 2 mo
**En-masse retraction**	**Hwang et al** 2017 Korea Parallel Retrospective	28 (23F/5M), split into two equal groups (14 + 14)	Mean 22.2 (19–29)	2 arms Conventional orthodontics after maxillary 1st-premolar extractions vs Orthodontics + ASO after the same extractions	Conventional (system not specified)	Not reported	Not reported	extraction of the maxillary first	Not reported	Maxillary anterior ASO; labial vertical incisions at premolars + midline; tunneling; osteotomy to piriform aperture; horizontal palatal osteotomy	No mm values provided; course of osteotomies described as above	Modified Wassmund (Lee’s) with preserved blood supply to labial/palatal mucosa	Not reported	Not reported	Single	Two time points: pre-treatment (T0) and end of active treatment (T1)	Shorter with ASO: ASO ≈ 22.05 months vs Conventional≈28.2 months
	**Singh & Jayan** 2019 India Parallel RCT	30 (NR)	18–40	**2 arms:** PAOO + orthodontics vs Conventional orthodontics	Roth Mini-Taurus	NR	NiTi coil springs	Likely premolars (not detailed)	TADs	**Labial only** (canine-to-canine, both arches)	Not specified (vertical + horizontal labial cuts canine–canine)	Corticotomy + DFDBA	Yes	Bur + graft	Single	Until debonding	PAOO: 12.7 ± 1.3 mo vs Ctrl: 21.3 ± 2.0 mo
**Presurgical decompensation**	**Ahn et al** 2016 Korea SMD Retrospective	30 (15 + 15)	Exp: 23.1 ± 6.2; Ctrl: 21.5 ± 3.3	**2 arms:** Experimental (Aug corticotomy) vs Control (Conventional presurgical)	Roth 0.022	0.016 × 0.022 NiTi	NR	Not reported	Conventional	**Labial only** (mandibular anteriors)	Vertical 2–3 mm below crest → 2–3 mm below apex, connected by a horizontal cut	Vertical + horizontal cuts, Bio-Oss	Yes	Bur or piezo	Single	8–11 mo	Exp: 8.7 mo vs Ctrl: 10.9 mo
	**Ma et al** 2023 China Parallel Prospective CCT	36 (21F/15M)	17–26 (mean ~21)	**2 arms:** Aug corticotomy-assisted vs Conventional	Roth fixed 0.022	0.014 → 0.018 × 0.025 NiTi → 0.018 × 0.025 SS	NR	Max 1st premolars extracted	Conventional	**Labial only** (mandibular incisors)	Not specified	Piezosurgical vertical cuts + Bio-Oss + membrane	Yes	Piezosurgery	Single	15–23 mo	AC: 15.7 ± 4.1 mo vs Ctrl: 23.3 ± 4.5 mo
	**Wang et al** 2013 China Parallel Prospective CCT	56 (26 corticotomy, 30 control)	Mean ~23–25 (SD ~ 3–4)	**2 arms:** Aug corticotomy-assisted vs Conventional	Fixed orthodontic appliance (details not specified)	0.016, 0.018, 0.018 × 0.025, 0.019 × 0.025 NiTi → 0.019 × 0.025 SS	Not specified	Non-extraction (mild crowding ≤3 mm)	Not specified	**Labial only** (mandibular anterior region)	Vertical grooves 1–2 mm below crest to 2–3 mm beyond apex, full-thickness flap	Labial flap, selective corticotomy + bone graft + membrane	Yes	Bur + membrane	Single	Until completion of presurgical orthodontics	G1: 7.8 ± 4.2 mo; G2: 13.3 ± 3.5 mo

**Abbreviations are listed in alphabetical order: AC**: Augmented corticotomy; **ACAO**: Alveolar corticotomy-assisted orthodontics; **ASO**: Anterior segmental osteotomy; **CBCT**: Cone-beam computed tomography; **CCT**: Controlled clinical trial; **Ctrl**: Control; **DFDBA**: Demineralized freeze-dried bone allograft; **Exp**: Experimental; **HANT**: Heat-activated nickel-titanium; **MBT**: McLaughlin–Bennett–Trevisi prescription; **mo**: months; **NiTi**: Nickel–titanium; **NR**: Not reported; **PAOO**: Periodontally accelerated osteogenic orthodontics; **RCT**: Randomized controlled trial; **SMD**: Split-mouth design; **SS**: Stainless steel; **TADs**: Temporary anchorage devices; **TPA**: Transpalatal arch

**Table 3:** Characteristics of root resorption assessment in the included studies.

Treatment goal	Author / Setting	Jaw / Teeth	Timing	Primary/ Secondary	Assessment method	Measurement	Analysis	Findings
**Alignment & leveling**	**Alam et al. / 2025** / Saudi Arabia	Both arches, multiple teeth	Baseline & post-treatment	Primary	CBCT	Not specified (mm)	t-test	ACAO: 0.8 mm vs Ctrl: 0.5 mm; NS clinically
	**Ferguson et al**/ 2016/ UAE + USA	Maxillary central incisors	T1 vs T2 (before and after treatment)	Primary	Periapical	Tooth length (mm)	t-test	PAOO –0.3 mm vs Conv –0.7 mm; lower measured root shortening was reported in the PAOO group
**Canine retraction**	**Abbas et al** / 2016 / Egypt /	Maxillary canines after first premolar extraction	Baseline, then every 2 weeks across 6 time points (3 months)	Secondary	CBCT	Not specified (mm)	paired t-tests (within groups)	Lower measured RR was reported on the surgical side than on the control side
	**Baiomy et al**/2021 / Egypt	Maxillary canines	Baseline & after retraction	Secondary	Periapical & panoramic	Not specified (mm)	Paired/unpaired t	G I: –0.3 mm; G II: –0.4 mm; Ctrl: –0.3 mm; NS
	**Raza et al**/ 2021/ India	Maxillary canines	T0 & post-retraction (~7 mo)	Secondary	CBCT	Tooth length (mm) (cusp–apex)	t-test	Test 0.24 mm vs Ctrl 0.53 mm; lower measured RR was reported in the test group
	**Sharma et al**/ 2020/ India	Maxillary canines	Before & after retraction (~6–7 mo)	Secondary	Periapical (Malmgren index)	Malmgren index, Grading (0–4)	t-test, Chi²	No significant RR in either group
**En-masse retraction**	**Hwang et al**/ 2017/ Korea	Both arches anterior segment	Two points: pre-treatment (T0) and end of active treatment (T1)	Primary	CBCT	Tooth length (mm)	Mann–Whitney U	The ASO group showed lower measured RR in this CBCT-based comparison
	**Singh & Jayan** / 2019/ India	Both arches, anterior teeth	Baseline & post-treatment	Secondary	Periapical (Sharpe index)	Categorical scoring	Mann–Whitney, Wilcoxon	No significant difference between PAOO vs Conv
**Presurgical decompensation**	**Ahn et al**/ 2016 / Korea	Mandibular anteriors	Baseline & post-decompensation	Secondary	CBCT	Root length + alveolar metrics	t-test, ANOVA	Both ~–0.6 to –0.8 mm; no difference
	**Ma et al**/ 2023/ China	Mandibular central incisors	T0 vs T1 (pre vs post presurgical phase)	Secondary	CBCT	Root length (mm)	t-test	Both groups ~ –1 mm; no difference
	**Wang et al** /2013/ China	Mandibular incisors	T0 vs T1 (pre vs post presurgical phase)	Secondary	CBCT + cephalograms	Root length (mm)	t-tests	Less measured root shortening was reported in the corticotomy group

**Abbreviations are listed in alphabetical order: ANOVA**: Analysis of variance; **ASO**: Anterior segmental osteotomy; **CBCT**: Cone-beam computed tomography; **Chi**²: Chi-squared test; **Conv**: Conventional treatment; **Ctrl**: Control (group); **G**: Group; **mm**: millimeter; **mo**: months; **NS**: Not significant; **PAOO**: Periodontally accelerated osteogenic orthodontics; **RR**: Root resorption; **T0**: Baseline (pre-treatment) time point; **T1**: First follow-up/time point; **T2**: Second follow-up/time point; **T3**: Third follow-up/time point**; t-test**: Student’s t-test.

### Risk of bias and quality assessment of RR in the included studies

**Fig 2:**
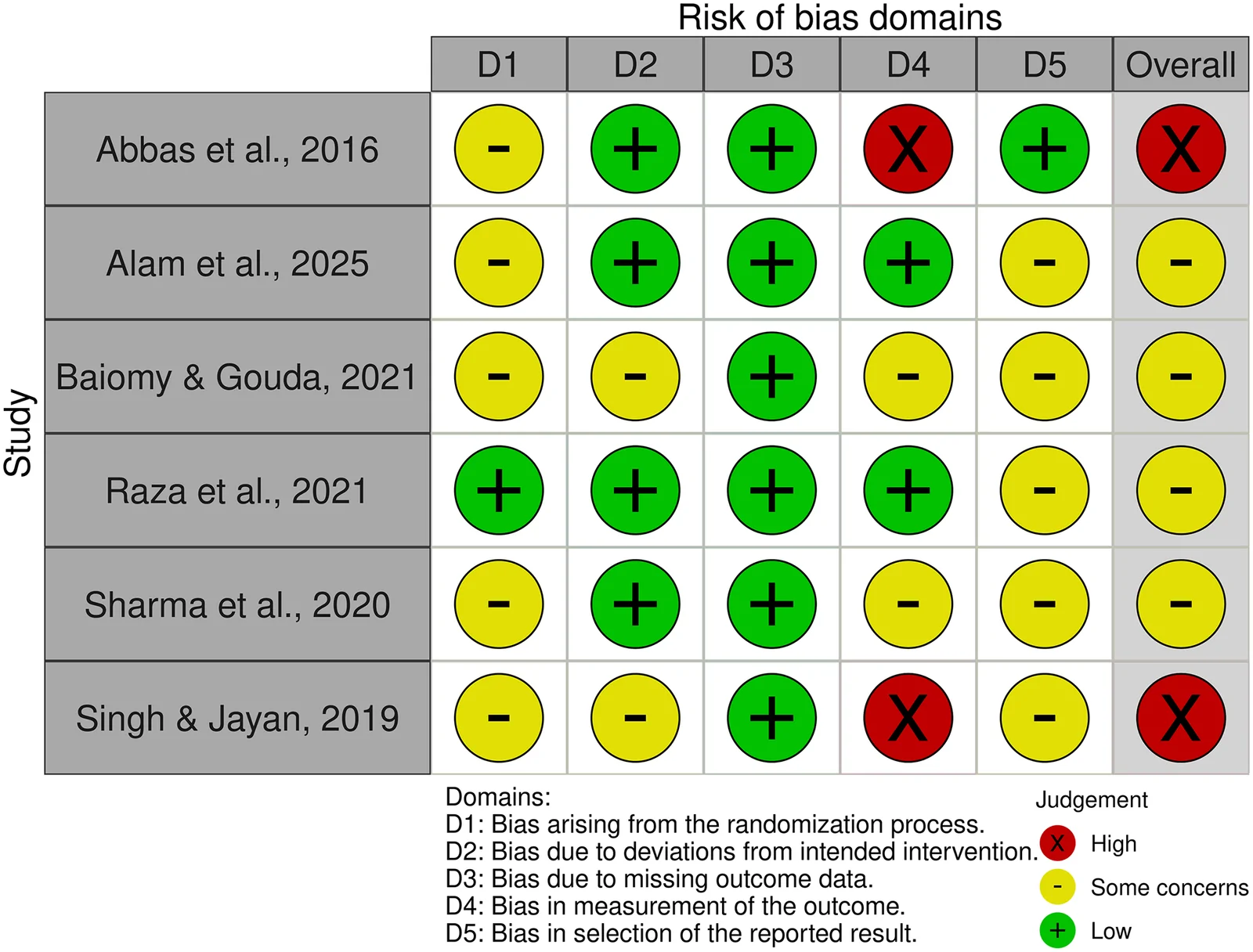
Risk of bias summary of RCTs: the review authors’ judgments about each item of the risk of bias for the included studies using the RoB 2 tool. Source: Created by the authors.

**Fig 3:**
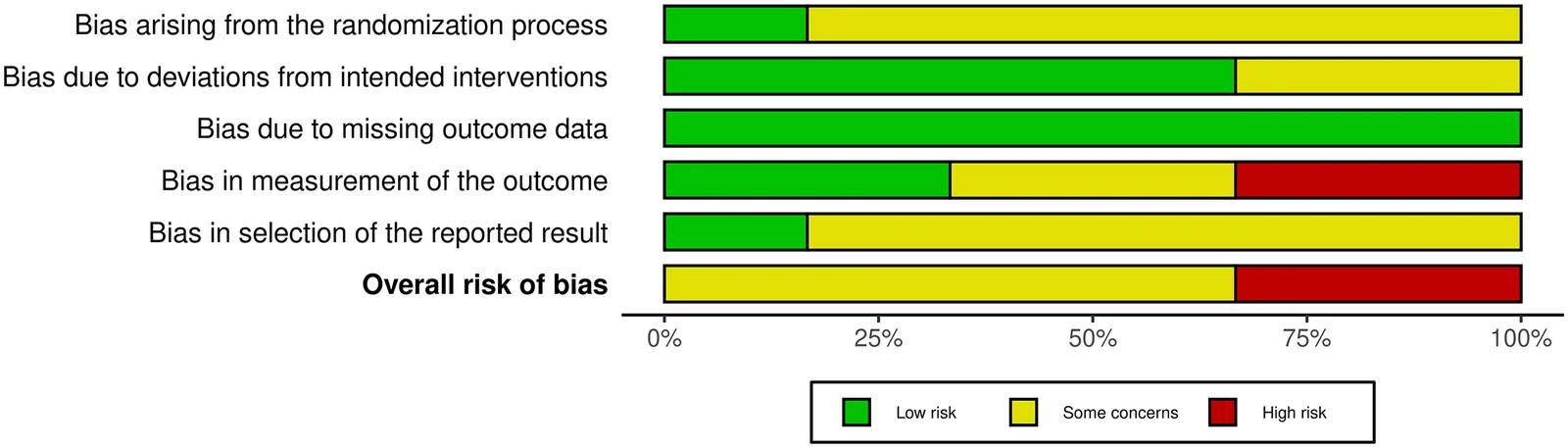
The overall risk of bias score for each field of RCTs: the review authors’ judgments about each item of the risk of bias, presented as percentages across all the studies included. Source: Created by the authors.

Four of six RCTs showed some concerns, and two showed a high risk of bias. Figs 2 and 3 summarize these ratings using the RoB 2 tool. The main sources of “some concerns” were randomization and selection of the reported result, each affecting 83% of RCTs, while high risk stemmed primarily from issues in outcome measurement, observed in 33% of RCTs. Supporting reasons are detailed in S4 Table.

**Fig 4:**
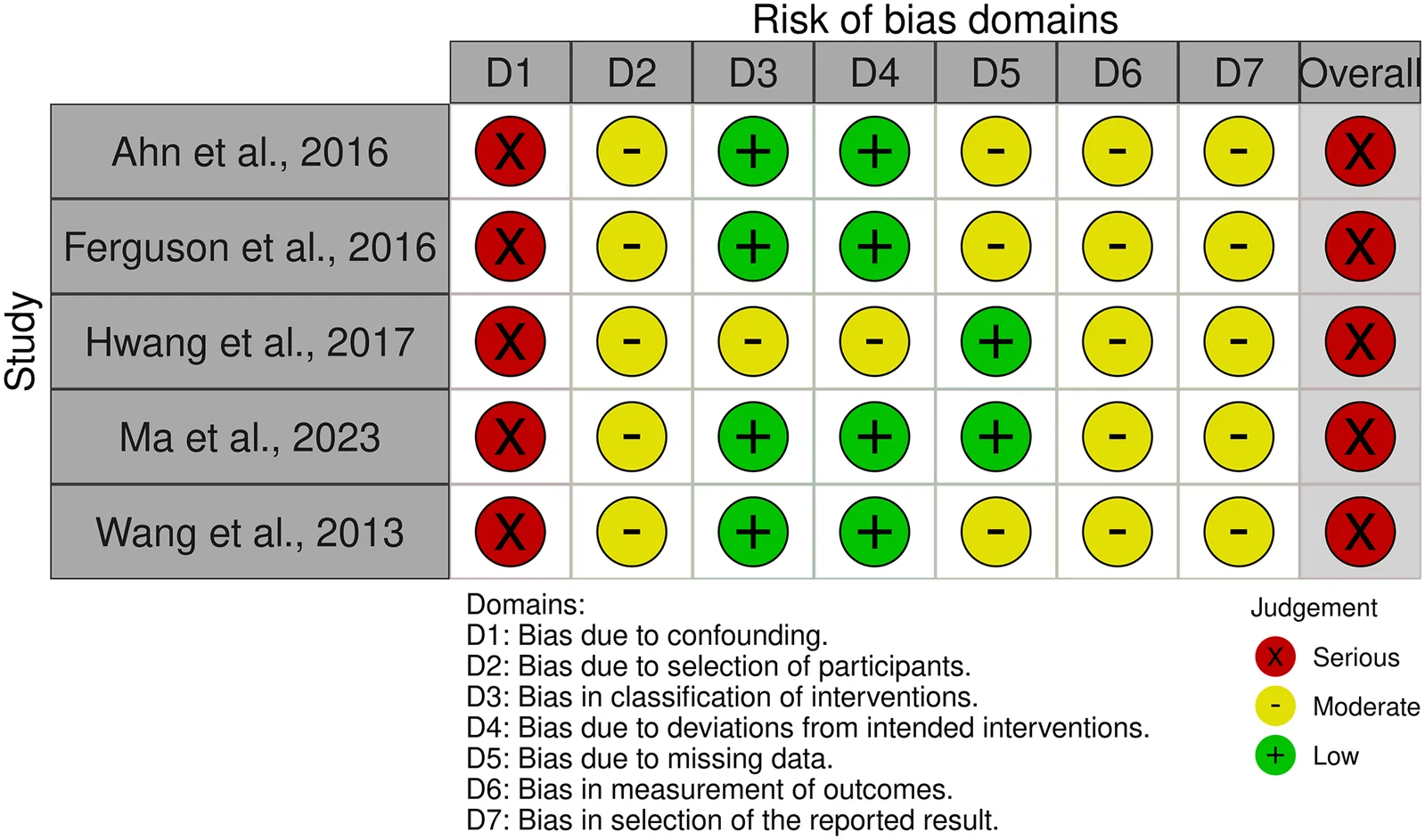
Risk of bias summary of CCTs: the review authors’ judgments about each item of the risk of bias for the included studies using the ROBINS-I tool. Source: Created by the authors.

**Fig 5:**
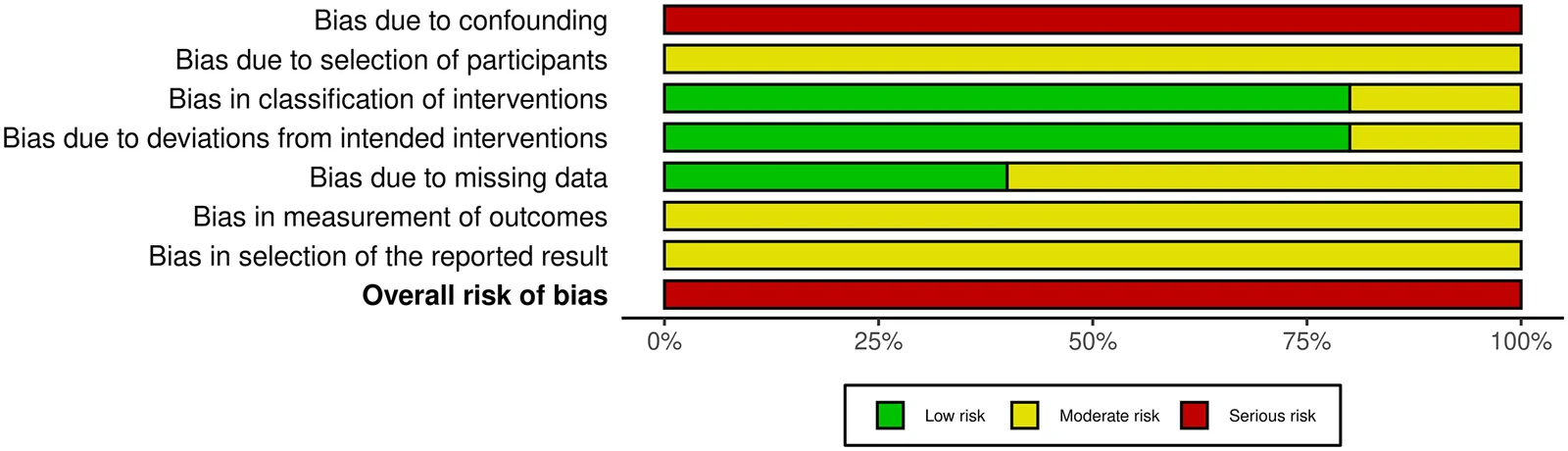
The overall risk of bias score for each field of CCTs: the review authors’ judgments about each item of the risk of bias, presented as percentages across all the studies included. Source: Created by the authors.

All non-randomized studies were rated as high risk due to confounding in the first domain. Figs 4 and 5 present these findings using the ROBINS-I tool. A rationale is provided in S5 Table.

**Fig 6:**
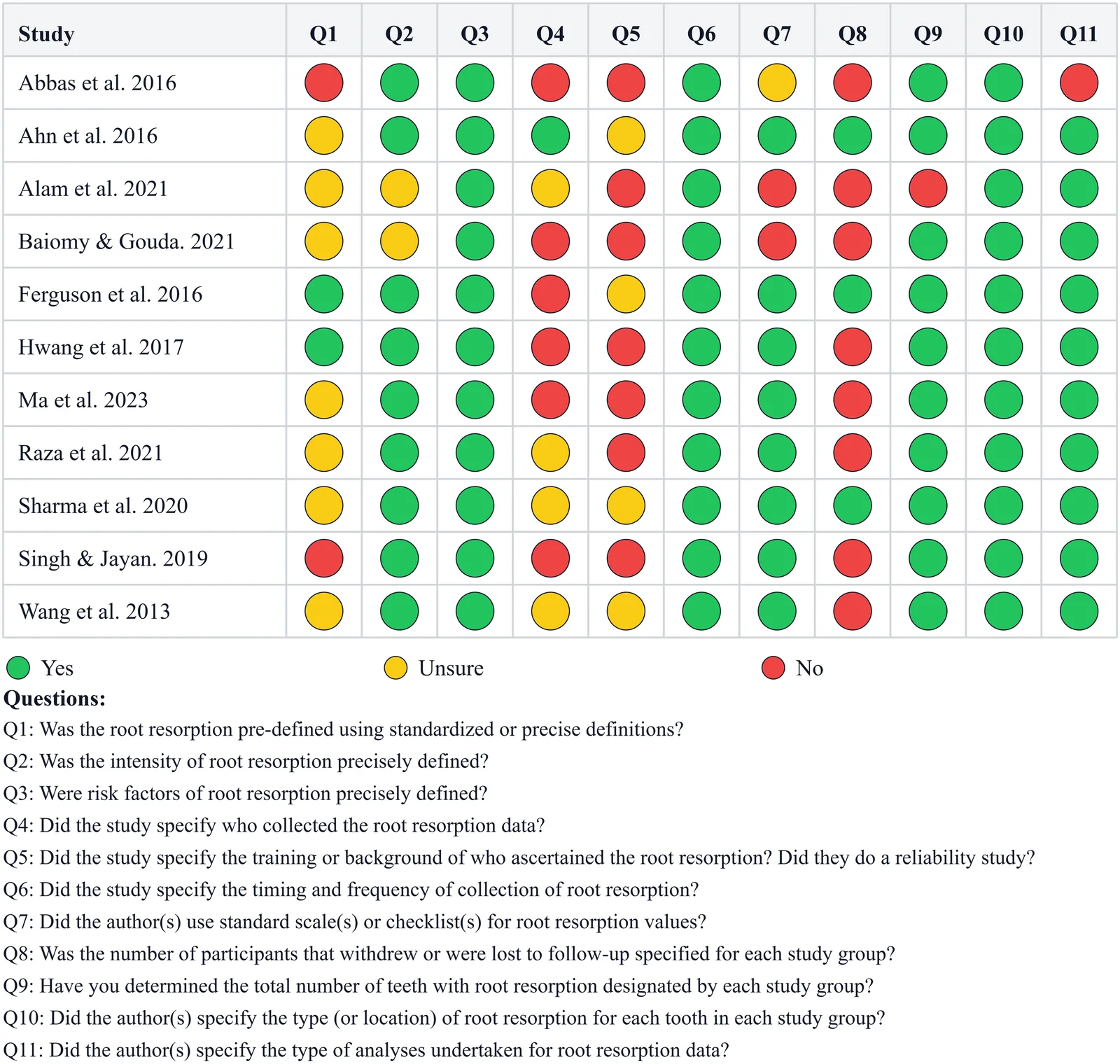
Summary of the quality for RR assessment in RCTs & CCTs: the review authors’ judgments about each question of the quality of RR assessment for the included studies using the McHarm tool. Source: Created by the authors.

**Fig 7:**
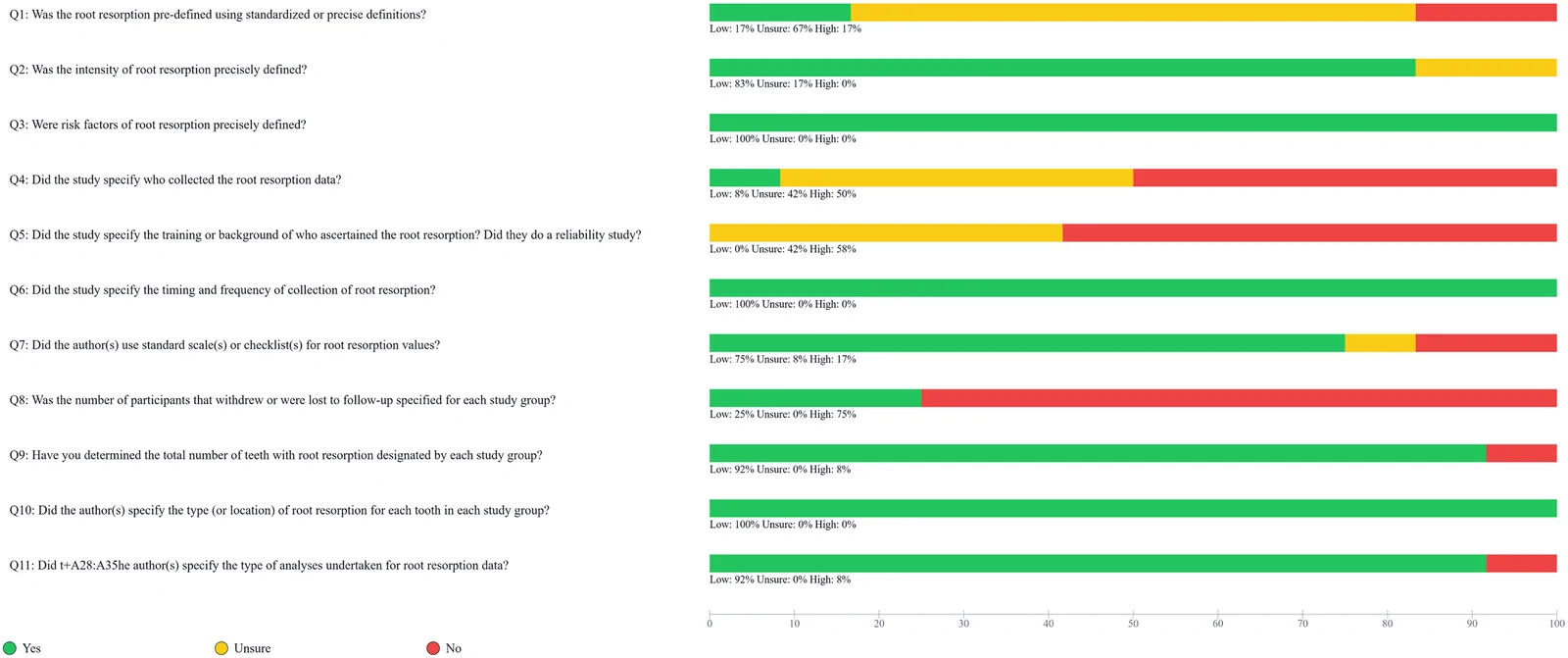
The quality for RR assessment score in each domain of RCTs & CCTs: the review authors’ judgments about each question of the quality of RR assessment, presented as percentages across all the studies included. Source: Created by the authors.

Assessment of RR quality using the modified McHarm tool revealed high risk in 73% of cases for not specifying withdrawals or losses to follow-up by group, 64% for insufficient assessor training, and 55% for not identifying data collectors. Additionally, 64% showed some concerns due to unclear definitions of RR. Figs 6 and 7 (created via HarmsVis, MRS Edition [36]), and S6 Table provide further details.

### Main findings of the effect of acceleration on RR

The impact of ISAO on RR was examined across four orthodontic objectives: leveling and alignment, canine retraction, en-masse retraction, and presurgical decompensation. For each objective, quantitative outcomes (means ± SD or ranges) were extracted, and between-group differences were interpreted with respect to both statistical significance and clinical relevance (Tables 4 and 5).

**Table 4:** Structured narrative summary of RR findings across treatment stages.

Treatment type	Site	RR measurement method	Surgical intervention	Observed between-group finding	Root resorption findings	Primary study	Analyzed units	P value
Alignment & leveling	Both arches	CBCT	ACAO (full flap, piezo)	Slightly higher measured RR in ACAO: 0.8 ± 0.3 mm vs 0.5 ± 0.2 mm (Δ≈+0.3 mm; clinically acceptable)	Slightly higher measured RR (statistically significant, but clinically small)	Alam et al (2025)	Not reported (per-tooth)	< 0.05
	Maxillary centrals	Periapical	PAOO (labial/lingual decortication + augmentation)	Slightly lower measured RR in PAOO: –0.3 mm vs –0.7 mm (Δ ≈ –0.4 mm)	Associated with slightly lower measured RR in the surgical group	Ferguson et al (2016)	107 teeth	0.03
Canine retraction	Maxilla	Periapical + panoramic	Vertical corticotomy vs modified (vertical + perforations)	No significant difference vs control (≈ –0.3 to –0.4 mm; NS)	**No difference**	Baiomy et al (2021)	48 teeth	> 0.05
		Periapical (Malmgren index)	Corticotomy distal to the canine	No clear between-group difference on the Malmgren index; both sides remained within grades 1–2.	**No difference**	Sharma et al (2020)	28 teeth	> 0.05
		CBCT	Corticotomy distal to the canine	Lower measured RR on the surgical (experimental) sides than on the control sides	Associated with slightly lower measured RR in the surgical group	Abbas et al (2016)	20 teeth	< 0.05
		CBCT	Corticotomy + perforations + graft	Lower measured RR on the test side: 0.24 ± 0.10 mm vs 0.53 ± 0.10 mm (Δ ≈ –0.29 mm)	Associated with slightly lower measured RR in the surgical group	Raza et al (2021)	20 teeth	< 0.05
En-masse retraction	Both arches	Periapical (Sharpe index)	PAOO + DFDBA	No treatment effect between PAOO and control; between-group comparison not significant (Mann-Whitney U = 105.5; Wilcoxon = 225.50, Z score = −0.342).	**No difference**	Singh & Jayan (2019)	360 teeth	0.732
		CBCT	ASO	Lower measured RR with ASO in this CBCT-based comparison: 0.93 mm (ASO) vs 1.61 mm (Conventional)	Associated with slightly lower measured RR in the surgical group	Hwang et al (2017)	198 teeth	< 0.01
Presurgical decompensation	Right Mand. incisor	CBCT	Class III high-angle: Augmented corticotomy + graft + membrane	No significant between-group difference in root-length change (1.16 ± 1.06 mm vs 0.82 ± 0.64 mm; p = 0.305).	**No difference**	Ma et al (2023)	144 teeth	P = 0.305
	Mand. incisors		Class III: Labial corticotomy + bone graft & membrane	Less measured root shortening was reported in the corticotomy group (–1.78 to –0.10 vs –1.96 to –0.76)	Associated with slightly lower measured RR in the corticotomy group	Wang et al (2013)	224 teeth	< 0.001
	Mand. anteriors		Augmented corticotomy + xenograft	~0.6–0.8 mm decrease in both; no significant difference	**No difference**	Ahn et al (2016)	180 teeth	> 0.05

**Abbreviations are listed in alphabetical order: ACAO**: Alveolar corticotomy-assisted orthodontics; **ASO**: Anterior segmental osteotomy; **CBCT**: Cone-beam computed tomography; **DFDBA**: Demineralized freeze-dried bone allograft; **Mand**.: Mandibular; **mm**: millimeter; **NS**: Not significant; **P**: p-value (probability); **PAOO**: Periodontally accelerated osteogenic orthodontics; **RR**: Root resorption.

**Table 5:** Summary of findings according to GRADE for the included studies.

Interventions	Quality assessment criteria							Summary of findings		
	Measurement method	No. of studies	Risk of bias	Inconsistency	Indirectness	Imprecision	Other considerations	No. of patients	Observed measured RR finding	Overall quality of evidence
**Root resorption in leveling and alignment studies**										
**Upper & Lower Jaws**			serious	not serious	not serious	Very serious	none	50	Slightly higher measured RR with ACAO: 0.8 ± 0.3 mm vs 0.5 ± 0.2 mm; mean difference ≈ +0.3 mm, p < 0.05; no severe RR reported	⨁◯◯◯^**a**^Very low
Using ACAO	CBCT	1 RCT Alam 2025 [13]								
**Upper Jaw regions**			Very serious	not serious	not serious	serious	none	54	Associated with slightly lower measured RR: MD ≈ −0.4 mm (0.3 mm vs 0.7 mm); both groups showed small absolute RR; P ≈ 0.03 between groups	⨁◯◯◯^**b**^Very low
Using PAOO	Periapical	1 CCT Ferguson 2016 [15]								
**Root resorption in retraction canine studies**										
Using Corticotomy	Periapical + panoramic, Root length (mm)	1 RCT Baiomy 2021 [30]	serious	not serious	not serious	serious	none	12	No clear between-group difference in measured RR. Group I: corticotomy ≈ −0.3 mm vs control ≈ −0.3 mm (p ≈ 0.92).	⨁⨁◯◯^**c**^low
	Periapical (Malmgren index)	1 RCT Sharma 2020 [32]	serious	not serious	not serious	serious	none	14	No clear between-group difference in measured RR was observed; both sides showed no clinically significant RR over the study period (IOPA, Malmgren/Levander index).	⨁⨁◯◯^**d**^low
	CBCT	1 RCT Abbas 2016 [29]	Very serious	not serious	not serious	serious	none	10	Lower measured RR was reported on the corticotomy side than on the contralateral control side over 3 months.	⨁◯◯◯^**e**^Very low
Using Corticotomy + perforation	Periapical + panoramic, Root length (mm)	1 RCT Baiomy 2021 [30]	serious	not serious	not serious	serious	none	12	No meaningful difference in RR. Group II: corticotomy + perforation≈ −0.4 mm vs control ≈ −0.3 mm (p ≈ 0.20).	⨁⨁◯◯^**f**^low
	CBCT	1 RCT Raza 2021 [31]	serious	not serious	not serious	serious	none	10	Lower measured RR was reported with corticotomy + perforations: MD ≈ −0.29 mm (0.24 vs 0.53 mm), P < 0.05	⨁⨁◯◯^**g**^low
**Root resorption in En-masse retraction studies**										
**Upper & Lower Jaws**			Very serious	not serious	not serious	serious	none	30	No clear between-group difference in measured RR between PAOO and control (Mann–Whitney P = 0.732); absolute RR scores are small in both groups	⨁◯◯◯^**h**^Very low
Using PAOO	Periapical (Sharpe index)	1 RCT Singh 2019 [28]								
Using ASO	CBCT	1 CCT Hwang 2017 [33]	Very serious	not serious	not serious	serious	none	28	Lower measured RR was reported in the ASO group than in the conventional orthodontic group in this retrospective CBCT-based comparison (0.93 ± 0.62 mm vs 1.61 ± 0.45 mm; p = 0.006).	⨁◯◯◯^**i**^Very low
**Root resorption in Presurgical decompensation+**										
**Right Mand. incisors**			Very serious	not serious	not serious	serious	none	36	No clear increase in measured RR was observed with AC compared with control during the presurgical phase (authors’ conclusion based on CBCT root-length change).	⨁◯◯◯^**j**^Very low
Using Augmented corticotomy + graft + membrane	CBCT	1 CCT Ma 2023 [14]								
**Mand. incisors**			Very serious	not serious	not serious	serious	none	56	A lower measured RR was reported in the AC group during the presurgical phase (group means indicated less apical shortening with AC; between-group P < 0.001, as reported by the authors).	⨁◯◯◯^**k**^Very low
Using Augmented corticotomy + graft + membrane	CBCT	1 CCT Wang 2013 [34]								
**Mand. anterior**			Very serious	not serious	not serious	serious	none	30	No clear between-group difference in measured RR across central/lateral incisors and canines (e.g., central incisors: −0.59 mm [AC] vs −0.70 mm [control]; group comparison NS)	⨁◯◯◯^**l**^Very low
Using Augmented corticotomy + graft	CBCT	1 CCT Ahn 2016 [35]								

**Abbreviations are listed in alphabetical order: AC**: Augmented corticotomy; **ACAO**: Alveolar corticotomy-assisted orthodontics; **ASO**: Anterior segmental osteotomy; **CBCT**: Cone-beam computed tomography; **GRADE**: Grading of Recommendations, Assessment, Development and Evaluation; **II**: Class II malocclusion; **IOPA**: Intraoral periapical radiograph; **ISBR**: Inter-septal bone reduction; **MD**: Mean difference; **NS:** Not significant; **PAOO**: Periodontally Accelerated Osteogenic Orthodontics; **RCT**: Randomized controlled trial; **RR**: Root resorption.

a Decline one level for risk of bias [13] (some concern in the randomization process, and selection of the reported result), and two levels for imprecision***

b Decline two levels for risk of bias [15] (some concern in the selection of participants, missing data, measurement of outcomes, and selection of the reported result, with high risk in confounding), and one level for imprecision***

c Decline one level for risk of bias [30] (some concern in the randomization process, Deviations from Interventions, Measurement of Outcomes, and selection of the reported result), and one level for imprecision***

d Decline one level for risk of bias [32] (some concern in the randomization process, Deviations from Interventions, Missing Outcome Data, Measurement of Outcomes, and selection of the reported result), and one level for imprecision***

e Decline two levels for risk of bias [29] (some concern in the randomization process, with high risk in Measurement of Outcomes), and one level for imprecision***

f Decline one level for risk of bias [30] (some concern in the randomization process, Measurement of Outcomes, and selection of the reported result), and one level for imprecision***

g Decline one level for risk of bias [31] (some concern in the selection of the reported result), and one level for imprecision***

h Decline two levels for risk of bias [28] (some concern in the randomization process, Deviations from Interventions, and selection of the reported result, with high risk in Measurement of Outcomes), and one level for imprecision***

i Decline two levels for risk of bias [33] (some concern in the selection of participants, classification of intervention, deviations from intended interventions, measurement of outcomes, and selection of the reported result, with high risk in confounding), and one level for imprecision***

j Decline two levels for risk of bias [14] (some concern in the selection of participants, measurement of outcomes, and selection of the reported result, with high risk in confounding), and one level for imprecision***

k Decline two levels for risk of bias [34] (some concern in the selection of participants, missing data, measurement of outcomes, and selection of the reported result, with high risk in confounding), and one level for imprecision***

l Decline two levels for risk of bias [35] (some concern in the selection of participants, missing data, measurement of outcomes, and selection of the reported result, with high risk in confounding), and one level for imprecision***

* Wide variance of point estimates across studies

** Indirectness: Short assessment duration (study time frame), and a difference between desired (patient-important outcomes) and measured outcomes.

*** Imprecision: Limited number of trials and sample size.

Because RR was assessed using non-equivalent imaging modalities and outcome metrics across the included studies, the following narrative synthesis is organized by treatment objective and interpreted within modality- and metric-specific strata. Absolute numerical differences, especially those expressed in millimeters, were not considered directly comparable across CBCT, two-dimensional radiography, and categorical scoring systems, or across markedly different follow-up periods.

### Leveling and alignment

In the CBCT-based corticotomy study, the surgical group showed slightly greater RR than the control group (0.8 ± 0.3 mm vs 0.5 ± 0.2 mm), corresponding to an approximate mean difference of 0.3 mm (p < 0.05; GRADE: very low) [13]. Although statistically significant, this difference was small in absolute terms.

In contrast, in the periapical radiography-based PAOO study of maxillary incisors, the surgical group showed less root shortening than the control group (approximately 0.3 mm vs 0.7 mm), corresponding to an approximate mean difference of 0.4 mm in favor of surgery (p = 0.03; GRADE: very low) [15].

These findings should not be interpreted as directly comparable effect sizes, because the studies differed in imaging modality, outcome measurement framework, and design. Therefore, within leveling and alignment, the available evidence suggests only small, context-specific differences in RR, rather than a consistent directional effect across interventions.

### Canine retraction

In the periapical/panoramic root-length study, neither vertical corticotomy nor modified corticotomy with perforations showed a significant difference in RR compared with the control side, with small absolute changes of approximately 0.3–0.4 mm (p > 0.05; GRADE: low) [30].

Similarly, in the periapical radiography study using the Malmgren index, shifts remained within grades 1–2 and were comparable between the corticotomy and control sides, indicating no clear between-group difference on this categorical scale (p > 0.05; GRADE: low) [32].

By contrast, in the CBCT-based split-mouth study, a posterior corticotomy placed distal to the canine was associated with lower RR on the surgical side than on the contralateral control side (p < 0.05; GRADE: very low), although the absolute magnitude was not numerically tabulated in the report [29].

In another CBCT-based study, a protocol combining corticotomy, perforations, and grafting showed lower mean RR in the surgical group than in the control group (0.24 ± 0.10 mm vs 0.53 ± 0.10 mm), corresponding to an approximate mean difference of 0.29 mm in favor of surgery (p < 0.05; GRADE: low) [31].

Because these studies differed in imaging modality, outcome metric, and protocol design, their numerical results should not be interpreted as directly comparable effect sizes. Accordingly, the canine-retraction evidence is best read as showing no clear harm signal in two-dimensional or index-based assessments of non-augmented corticotomy, whereas CBCT-based studies suggest slightly lower measured RR in some site-specific or augmented protocols, rather than a confirmed reduction in biologic risk.

### En-masse retraction

In the PAOO study with demineralized freeze-dried bone allograft (DFDBA), no statistically significant difference in RR was detected between the surgical and control groups (p = 0.732; GRADE: very low) [28].

In the anterior segmental osteotomy (ASO) study, root resorption was lower in the surgical group than in the conventional treatment group (0.93 mm vs 1.61 mm), corresponding to an approximate mean difference of 0.68 mm in favor of ASO (p < 0.01; GRADE: very low) [33].

However, these findings should not be interpreted as directly comparable effect sizes, because the studies differed in surgical protocol, study design, measurement framework, and follow-up context. Accordingly, the en-masse retraction evidence is best interpreted as showing no clear between-group difference in measured RR in the available PAOO comparison, whereas a separate non-randomized ASO study was associated with lower measured RR. Given the very low certainty of evidence and the methodological differences between studies, no uniform conclusion can be drawn across en-masse retraction protocols.

### Presurgical decompensation

In high-angle skeletal Class III cases treated with augmented corticotomy combined with graft and membrane, the mean change in root length did not differ significantly between the surgical and control groups (1.16 ± 1.06 mm vs 0.82 ± 0.64 mm; p = 0.305; GRADE: very low) [14].

In a separate study of labial corticotomy combined with bone grafting and membrane coverage, the surgical group showed less root shortening than the control group (0.10–1.78 mm vs 0.76–1.96 mm; p < 0.001; GRADE: very low) [34].

In the xenograft-augmented corticotomy study, root length decreased by approximately 0.6–0.8 mm in both groups, with no significant between-group difference (p > 0.05; GRADE: very low) [35].

However, these findings should not be interpreted as directly comparable effect sizes, because the studies differed in augmentation design, measurement framework, follow-up context, and underlying presurgical mechanics. Accordingly, the presurgical decompensation evidence is best interpreted as showing no consistent increase in measured RR with augmented approaches, while isolated protocols were associated with less measured root shortening in specific clinical contexts. Given the very low certainty of evidence and the methodological heterogeneity across studies, no uniform conclusion can be drawn across presurgical decompensation protocols.

## Discussion

Orthodontically induced root resorption (RR) is a common, force-dependent adverse effect, but its risk profile in invasive surgically assisted orthodontics (ISAO) is often obscured by heterogeneous imaging and selective harm reporting. A harm-focused framework helps address this. The use of McHarm, together with contemporary guidance such as CONSORT-Harms 2022, encourages clearer definition and reporting of RR (predefined outcomes, denominators, time-at-risk, severity grading, and imaging-related adverse events), thereby reducing under-ascertainment and outcome-reporting bias [21]. Because measurement determines inference, RR signals in this review were interpreted with respect to modality sensitivity. The methods used to assess RR were not uniform across the included studies. Seven studies used CBCT, three used periapical radiography, and one used a combined periapical/panoramic approach; in addition, the outcome was variably expressed either as linear root-length change in millimeters or by categorical indices such as the Malmgren or Sharpe scales, and some reports did not describe the measurement protocol in sufficient detail. This lack of standardization is important because measurement accuracy is modality-dependent. Recent evidence suggests that both periapical radiography and CBCT can identify external apical root resorption with acceptable diagnostic performance, although CBCT is generally more suitable for sensitive three-dimensional quantification, may better detect subtle changes under broader diagnostic thresholds, and is less affected by anatomic overlap than two-dimensional radiographs [5,37]. By contrast, panoramic imaging may underestimate the amount of root resorption when compared with CBCT [6]. Therefore, RR outcomes across the included studies cannot be considered fully comparable in either method or accuracy, and small between-study differences—particularly when derived from mixed or two-dimensional imaging methods—should be interpreted cautiously. The amount of force applied was not standardized across the included studies, and reporting was inconsistent across treatment objectives; while the four canine-retraction trials used relatively comparable 150 g NiTi coil mechanics, several alignments, en-masse retraction, and presurgical decompensation studies reported force only qualitatively or did not quantify it clearly at all [14,15,28,34]. Appliance systems and mechanics were likewise heterogeneous, spanning edgewise, Roth, MBT, and incompletely described fixed appliances, with additional variation in anchorage strategy, archwire progression, extraction timing, and movement objectives [14,15,28,34]. This variability is clinically relevant because recent evidence syntheses identify appliance type, movement type, extractions, treatment time, and force magnitude as important mechanical contributors to external apical root resorption [38]. The same contemporary evidence indicates that continuous forces, greater force magnitude, and longer treatment duration are associated with greater resorption risk, whereas intrusion concentrates stress apically and is considered particularly relevant to RR development [38,39]. Recent CBCT-based evidence also suggests that appliance design itself may influence the distribution of root resorption, as greater lower-incisor resorption was observed with lingual than with labial fixed appliances in a randomized trial [40]. Accordingly, some of the between-study differences observed in this review may reflect differences in force delivery and biomechanical setup, rather than the surgical adjunct alone, and this should be considered when interpreting discordant RR findings across protocols [38,40].

Within this framework, small, procedure-specific differences across corticotomy, PAOO, and segmental approaches should be interpreted against biologic plausibility (regional acceleratory phenomenon and altered force–time profiles) and the broader evidence that RR risk depends mainly on force magnitude, treatment duration, and tooth susceptibility rather than surgery itself [41,42]. This harm-focused review is the first to apply McHarm to RR in ISAO and therefore provides a methodological benchmark for future safety evaluations.

An important limitation of between-study interpretation is that RR was not measured within a uniform analytical framework. CBCT-derived linear changes, periapical tooth- or root-length measurements, and ordinal indices such as the Malmgren and Sharpe scales do not represent directly interchangeable outcomes. Likewise, follow-up periods ranged from short-term retraction assessments to full treatment or presurgical phases lasting many months, and the surgical protocols themselves differed in extent, grafting, instrumentation, and biologic intent. Therefore, numerical differences reported across studies—particularly absolute millimeter changes—should be interpreted only within broadly similar modality, metric, and follow-up contexts, rather than as directly comparable effect sizes across the entire evidence base

### Alignment and leveling

During leveling and alignment, the apparent difference between plain corticotomy and graft-augmented PAOO may reflect both biologic and methodological variation across studies. In the trial by Alam et al. [13], corticotomy without augmentation was associated with a small but statistically significant increase in RR detected on CBCT during early leveling. This finding may reflect transient differences in apical stress distribution during the initial alignment phase, although the mechanism cannot be confirmed from the available data. Similar short-term CBCT findings after corticotomy or corticision have been reported in the literature and may support this pattern, although cross-study comparisons remain limited by methodological heterogeneity. In contrast, Ferguson et al. [15] suggested that grafted PAOO may alter the local biologic and mechanical environment in a way that is associated with slightly lower measured root shortening in this study; however, this finding should be interpreted cautiously given the small absolute difference, the use of periapical radiography, and the very low certainty of evidence.

Overall, plain corticotomy was associated with slightly higher measured RR within clinically acceptable limits in one CBCT-based study, whereas graft-augmented PAOO was associated with slightly lower measured root shortening in a separate periapical study. These findings should not be interpreted as directly comparable effect estimates or as evidence of a consistent biologic advantage of one protocol over another.

### Canine retraction

Across four trials [29–32], the effect of surgically assisted protocols on canine RR appears to depend on both the specific protocol and the method of measurement. In corticotomy performed without augmentation, studies using periapical root-length measurements [30] or the Malmgren index [32] generally report no significant differences in RR between test and control sides. This pattern is consistent with the limited sensitivity of categorical grading systems to submillimetric changes and indicates that these studies did not show evidence of higher measured RR with vertical or modified corticotomy alone. In contrast, when CBCT is used, and corticotomy is placed distal to the canine [29], the surgical side was associated with lower measured RR, although this difference may reflect both protocol-specific biomechanics and the greater sensitivity of CBCT to small root-length changes. Augmented protocols that combine corticotomy, micro-perforations, and grafting [31] were associated with a small difference in measured RR (mean difference ≈ 0.3 mm), but the absolute magnitude was limited and should be interpreted cautiously in view of possible measurement variability and reduced force-exposure time. Although these findings are biologically compatible with a regional acceleratory response and altered force distribution, the available studies do not allow firm mechanistic inferences. Overall, non-augmented corticotomy appears RR-neutral, whereas site-specific or augmented approaches were associated with slightly lower measured RR in some CBCT-based comparisons. However, the observed differences were small and may partly reflect the measurement framework and shorter force exposure rather than a direct biologic protective effect.

### En-masse retraction

In en-masse retraction, the lack of a measurable reduction in RR with PAOO plus demineralized freeze-dried bone allograft (DFDBA) [28], compared with the lower measured RR reported for anterior segmental osteotomy (ASO) [33], should be interpreted in light of differences in treatment mechanics, force-exposure time, and measurement sensitivity rather than as directly comparable treatment effects. In PAOO, continuous orthodontic mechanics are still used to retract the entire anterior segment, so any biologic effect related to accelerated turnover may remain modest within the overall period of apical loading; as a result, any effect of the regional acceleratory phenomenon is modest and can be easily masked when RR is evaluated only with lower-resolution, end-of-treatment measurements [28]. In contrast, ASO closes extraction spaces surgically, thereby reducing the duration of orthodontic loading, which may contribute to lower measured RR values in comparison with conventional mechanics. When RR is assessed with CBCT, which more reliably detects submillimetric root-length changes than periapical radiographs, this difference may become more readily detectable [33]. Both approaches are consistent with RAP biology, but only ASO combines RAP with a meaningful reduction in force exposure, which likely explains the divergence observed among en-masse retraction protocols. Even so, this interpretation remains provisional because the supporting evidence is limited, heterogeneous, and at very low certainty.

### Presurgical decompensation

During presurgical decompensation, the RR pattern appears to be influenced more by augmentation design and imaging modality than by the surgical procedure itself. In high-angle Class III cases treated with augmented corticotomy plus graft and membrane, root-length changes did not differ significantly from controls, whereas alveolar support improved and dehiscence decreased, suggesting that labial decompensation can be performed without evidence of a higher measured RR signal within a reinforced alveolar housing [14]. Labial corticotomy combined with graft and membrane was associated with less measured root shortening than conventional mechanics in that study, but this finding should be interpreted cautiously because it may reflect shorter force exposure, measurement context, or both, rather than a direct biologic reduction in resorption susceptibility [34]. Xenograft-augmented protocols showing similar root-length reduction in test and control groups are best considered RR-neutral, as they were associated with similar measured root-length change to controls over short follow-up periods [35]. These findings are consistent with differences in augmentation design, corticotomy location, and imaging sensitivity (CBCT vs conventional indices). Although the certainty of evidence remains very low, no clear signal of disproportionately higher measured RR has emerged for augmented presurgical decompensation.

### Limitations

This harm-focused review was strengthened by a comprehensive search without language restrictions, explicit eligibility criteria, and a structured narrative synthesis organized by treatment objective. In addition, RR outcomes were appraised not only with conventional risk-of-bias tools (RoB 2 and ROBINS-I) but also with the modified McMaster Harms (McHarm) tool, which improved the consistency and transparency of harms assessment. Nevertheless, the conclusions are constrained primarily by limitations in the underlying evidence base. The number of eligible studies was small, sample sizes were modest, and many studies were affected by methodological limitations, including concerns or high risk of bias, confounding, and incomplete reporting of key design features. Follow-up was often short or uneven, which limits confidence in the stability and clinical relevance of the observed RR findings over time. Substantial clinical and methodological heterogeneity across surgical protocols, treatment objectives, imaging modalities, timing of outcome assessment, and RR definitions and measurement approaches further limited comparability and precluded a robust quantitative synthesis. Importantly, RR and other adverse effects were frequently secondary outcomes rather than prespecified primary endpoints, and harms were often assessed with non-standardized indices or reported without sufficient detail on withdrawals, assessor training, outcome definitions, or ascertainment procedures. As a result, some relevant limitations were not consistently acknowledged in the primary reports themselves, and both the magnitude and direction of the reported effects may have been influenced by measurement and reporting shortcomings. In addition, because only a limited number of heterogeneous studies were available, publication bias and small-study effects could not be meaningfully explored. Accordingly, the findings of this review should be interpreted cautiously and viewed as hypothesis-generating rather than definitive. Future research should prioritize adequately powered, prospectively designed trials with standardized RR outcomes, justified CBCT use, longer follow-up, and more complete and transparent harms reporting.

## Conclusions

Current low- to very low-certainty evidence does not demonstrate a large or consistent increase in RR with ISAO compared with conventional mechanics. However, the available studies are small, methodologically heterogeneous, and frequently at high risk of bias, which prevents firm conclusions about safety or about the relative effects of specific ISAO protocols. Across treatment objectives, most corticotomy-based protocols were associated with either similar measured RR or small differences that generally remained within commonly accepted clinical limits, but these findings should be interpreted cautiously. In some studies, graft-augmented corticotomy or osteotomy protocols were associated with similar or slightly lower measured RR; however, these signals were small, method-dependent, and hypothesis-generating rather than definitive.

For clinicians, these findings should not be interpreted as proof of safety. When ISAO is used, treatment planning should continue to emphasize individualized case selection, careful biomechanics and force control, preservation of the alveolar housing, and routine radiographic monitoring. This review also highlights the value of the modified McMaster Harms (McHarm) tool and structured CBCT-based assessment for improving harms evaluation. Larger, better-designed studies with standardized RR outcomes, clearer reporting of harms, and longer follow-up are required before firm conclusions can be drawn for specific protocols.

## Supporting information

S1 Table Electronic search strategy. (DOCX)

S2 Table Domains and judgments of the RoB 2, ROBINS-I, and McHarm tools. (DOCX)

S3 Table Studies excluded and reasons for exclusion. (DOCX)

S4 Table Risk of bias of the included RCTs in this systematic review, with supporting reasons. (DOCX)

S5 Table Risk of bias of the included CCTs in this systematic review, with supporting reasons. (DOCX)

S6 Table McMaster Quality Assessment Scale of root resorption of the included RCTs and CCTs in this systematic review, with supporting reasons. (DOCX)

## Abbreviations

ASO: Anterior Segmental Osteotomy
CBCT: Cone Beam Computed Tomography
CCT: Controlled Clinical Trial
DFDBA: Demineralized Freeze-Dried Bone Allograft
GRADE: Grading of Recommendations Assessment, Development, and Evaluation
ISAO: Invasive Surgically Accelerated Orthodontics
McHarm: McMaster University Harms Scale / Harms Assessment Tool
MISAO: Minimally Invasive Surgically Accelerated Orthodontics
OPG: Orthopantomogram (Panoramic Radiograph)
PAOO: Periodontally Accelerated Osteogenic Orthodontics
PICOS: Population, Intervention, Comparison, Outcome, Study Design
PRISMA: Preferred Reporting Items for Systematic Reviews and Meta-Analyses
PRISMA-Harms: PRISMA Harms Extension for Reporting Harms in Systematic Reviews
PROSPERO: International Prospective Register of Systematic Reviews
RAP: Regional Acceleratory Phenomenon
RCT: Randomized Controlled Trial
RoB 2: Revised Cochrane Risk of Bias Tool for Randomized Trials
ROBINS-I: Risk of Bias in Non-randomized Studies of Interventions
RR: Root Resorption
