# Supplementary material for: Does invasive surgical acceleration of orthodontic tooth movement increase root resorption? A harms-focused systematic review
Source: PLoS One. 2026 Aug 19;21(8):e0356175. doi: 10.1371/journal.pone.0356175 (PMC13489456; doi:10.1371/journal.pone.0356175)
Supplement: Supplementary file 1 [file pone.0356175.s001.docx]

| **S1 Table.** Electronic search strategy | | |
| --- | --- | --- |
| NO | Database | Search strategy |
| **1** | **PubMed**  Publication Date:  from no limit Until 25-8-2023 | (orthodontic*[tiab] OR orthodontics[MeSH Terms]) AND ("surgically accelerated orthodontic*"[tiab] OR "surgically assisted orthodontic*"[tiab] OR corticotomy[tiab] OR "alveolar corticotomy"[tiab] OR "periodontally accelerated osteogenic orthodontics"[tiab] OR "augmented corticotomy"[tiab] OR "anterior segmental osteotomy"[tiab]) AND ("root resorption"[MeSH Terms] OR "root resorption"[tiab] OR "external apical root resorption"[tiab]) AND (flap*[tiab] OR invasive[tiab]) NOT (animals[mh] NOT humans[mh]). |
| **2** | **Web of Science**  Publication Date:  from no limit Until 25-8-2023 | TS=(orthodontic* OR orthodontics) AND TS=("surgically accelerated orthodontic*" OR "surgically assisted orthodontic*" OR corticotomy OR "alveolar corticotomy" OR "periodontally accelerated osteogenic orthodontics" OR "augmented corticotomy" OR "anterior segmental osteotomy") AND TS=("root resorption" OR "external apical root resorption") AND TS=(flap* OR invasive). Document types: Article, Clinical Trial. |
| **3** | **Scopus**  Publication Date:  from no limit Until 25-8-2023 | TITLE-ABS-KEY(orthodontic* OR orthodontics) AND TITLE-ABS-KEY("surgically accelerated orthodontic*" OR "surgically assisted orthodontic*" OR corticotomy OR "alveolar corticotomy" OR "periodontally accelerated osteogenic orthodontics" OR "augmented corticotomy" OR "anterior segmental osteotomy") AND TITLE-ABS-KEY("root resorption" OR "external apical root resorption") AND TITLE-ABS-KEY(flap* OR invasive). No limits on year or language. |
| **4** | **Embase**  Publication Date:  from no limit Until 25-8-2023 | ('orthodontics'/exp OR orthodontic*:ab,ti) AND ('surgically accelerated orthodontic*':ab,ti OR 'surgically assisted orthodontic*':ab,ti OR corticotomy:ab,ti OR 'alveolar corticotomy':ab,ti OR 'periodontally accelerated osteogenic orthodontics':ab,ti OR 'augmented corticotomy':ab,ti OR 'anterior segmental osteotomy':ab,ti) AND ('root resorption'/exp OR 'root resorption':ab,ti OR 'external apical root resorption':ab,ti) AND (flap*:ab,ti OR invasive:ab,ti) NOT ([animals]/lim NOT [humans]/lim). |
| **5** | **Cochrane Central Register of Controlled Trials (CENTRAL)**  Publication Date:  from no limit Until 25-8-2023  Search field:  title, abstract,  keywords | (orthodontic* OR orthodontics) in Title, Abstract, Keywords AND ("surgically accelerated orthodontic*" OR "surgically assisted orthodontic*" OR corticotomy OR "alveolar corticotomy" OR "periodontally accelerated osteogenic orthodontics" OR "augmented corticotomy" OR "anterior segmental osteotomy") AND ("root resorption" OR "external apical root resorption") AND (flap* OR invasive). No restrictions applied. |
| **6** | **Google Scholar**  Publication Date:  from no limit Until 25-8-2023 | "orthodontic" AND ("surgically accelerated orthodontic" OR "surgically assisted orthodontic" OR corticotomy OR "alveolar corticotomy" OR "periodontally accelerated osteogenic orthodontics" OR "augmented corticotomy" OR "anterior segmental osteotomy") AND ("root resorption" OR "external apical root resorption") AND (flap* OR invasive). First 200 results screened by title/abstract. |
| **7** | **Trip**  Publication Date:  from no limit Until 25-8-2023 | orthodontic:ti,ab AND ("surgically accelerated orthodontic" OR "surgically assisted orthodontic" OR corticotomy OR "periodontally accelerated osteogenic orthodontics" OR "anterior segmental osteotomy") AND ("root resorption" OR "external apical root resorption") AND (flap* OR invasive). Filters: clinical studies in humans. |
| **8** | **OpenGrey**  http://www.opengrey.eu/ | orthodontic AND (corticotomy OR "surgically accelerated orthodontic" OR "surgically assisted orthodontic" OR "anterior segmental osteotomy") AND "root resorption" AND (flap* OR invasive) in Title/Abstract. No limits on year or language. |
| **9** | **ProQuest PQDT Open** | TI(orthodontic OR orthodontics) AND AB(("surgically accelerated orthodontic" OR "surgically assisted orthodontic" OR corticotomy OR "alveolar corticotomy" OR "periodontally accelerated osteogenic orthodontics" OR "augmented corticotomy" OR "anterior segmental osteotomy") AND ("root resorption" OR "external apical root resorption") AND (flap* OR invasive)). Source type: dissertations and theses. |
