# Supplementary material for: Does invasive surgical acceleration of orthodontic tooth movement increase root resorption? A harms-focused systematic review
Source: PLoS One. 2026 Aug 19;21(8):e0356175. doi: 10.1371/journal.pone.0356175 (PMC13489456; doi:10.1371/journal.pone.0356175)
Supplement: Supplementary file 2 [file pone.0356175.s002.docx]

| **S2 Table.** The RoB 2.0’, ROBINS-I’ and MMcHarm’ tools domains and judgments | | |
| --- | --- | --- |
| **Domains** | **Judgments** | |
| **RoB 2.0 tool (For RCTs)** | | |
| 1. Bias arising from the randomization process | **overall** | **Low, Some**  **concerns, High** |
| 2. Bias due to deviations from intended interventions |  |  |
| 3. Bias due to missing outcome data |  |  |
| 4. Bias in measurement of the outcome |  |  |
| 5. Bias in selection of the reported result |  |  |
| **ROBINS-I tool (For CCTs)** | | |
| 1. Bias due to confounding | **overall** | **Low, Moderate, Serious, Critical, No information** |
| 2. Bias in the selection of participants into the study |  |  |
| 3. Bias in the classification of interventions |  |  |
| 4. Bias due to deviations from intended interventions |  |  |
| 5. Bias due to missing data |  |  |
| 6. Bias in measurement of outcomes |  |  |
| 7. Bias in selection of the reported result |  |  |
| **MMcHarm tool (For RCTs & CCTs)** | | |
| 1. Was the root resorption **pre-defined** using standardized or precise definitions? | **Yes, No, Unsure** | |
| 2. Was **intensity** of root resorption precisely defined? | **Yes, No, Unsure** | |
| 3. Were **risk factors** of root resorption precisely defined? | **Yes, No, Unsure** | |
| 4. Did the study specify **who** collected the root resorption data? | **Yes, No, Unsure** | |
| 5. Did the study specify the **training** or **background** of who ascertained the root resorption? Did they do a **reliability study**? | **Yes, No, Unsure** | |
| 6. Did the study specify the **timing** and **frequency** of collection of root resorption? | **Yes, No, Unsure** | |
| 7. Did the author(s) use **standard scale**(s) or checklist(s) for root resorption values? | **Yes, No, Unsure** | |
| 8. Was the **number** of participants that withdrew or were lost to follow-up specified for each study group? | **Yes, No, Unsure** | |
| 9. Have you determined the **total number** of teeth with root resorption designated by each study group? | **Yes, No, Unsure** | |
| 10. Did the author(s) specify the **type** (or location) of root resorption for each tooth in each study group? | **Yes, No, Unsure** | |
| 11. Did the author(s) specify the type of **analyses** undertaken for root resorption data? | **Yes, No, Unsure** | |
| **RoB 2.0 tool: RCTs**: randomized clinical trials; **CCTs**: controlled clinical trials; **Low**: if all fields were estimated as "at low risk of bias"; **Some concerns**: if at least one domain was assessed as "some concerns" but not to be at "high risk of bias" for any domain; **High**: if at least one or more fields were estimated as "at high risk of bias" or if there were some concerns for multiple domains in a way that substantially lowered confidence in the result.  **ROBINS-I tool: Low**: if all fields were estimated as "at low risk of bias"; **Moderate**: if all domains were evaluated as "the low or moderate risk of bias"; **Serious**: if at least one domain assessed as "serious risk of bias" but not at critical risk of bias in any domain, **Critical**: if at least one domain assessed as "critical risk of bias"; **No information**: if there is no clear indication that the study is "at serious or critical risk of bias" and there is a lack of information in one or more key domains of bias.  **MMcHarm tool** :Modified McMaster University Harms scale; **Yes**: implying a less risk of bias; **No**: implying a high risk of bias; **intensity**: determine the incidence of harm, the units of measurement for the intensity of root resorption were: mm or mm3 or as a score; **Risk factors**: the appliance and intervention characteristics, the follow-up time, and the duration of treatment were determined as risk factors for root resorption that must be identified within the materials and methods section for each study. | | |
