# Supplementary material for: Does invasive surgical acceleration of orthodontic tooth movement increase root resorption? A harms-focused systematic review
Source: PLoS One. 2026 Aug 19;21(8):e0356175. doi: 10.1371/journal.pone.0356175 (PMC13489456; doi:10.1371/journal.pone.0356175)
Supplement: Supplementary file 3 [file pone.0356175.s003.docx]

| **S3 Table.** Studies excluded and reasons for exclusion | | | |
| --- | --- | --- | --- |
| 1. | Al-Naoum F, Hajeer MY, Al-Jundi A; Does alveolar corticotomy accelerate orthodontic tooth movement when retracting upper canines? A split-mouth design randomized controlled trial. J Oral Maxillofac Surg 2014;72(10):1880-9. doi: 10.1016/j.joms.2014.05.003. | Inadequate description of materials, methods, treatment groups, interventions, or outcomes regarding root resorption |  |
| 2. | Baeshen HA; The Effect of Partial Corticotomy on the Rate of Maxillary Canine Retraction: Clinical and Radiographic Study. Molecules 2020;25(20). doi: 10.3390/molecules25204837. |  |  |
| 3. | Fernandes L, Figueiredo DSF, Oliveira DD, et al.; The effects of corticotomy and piezocision in orthodontic canine retraction: a randomized controlled clinical trial. Prog Orthod 2021;22(1):37. doi: 10.1186/s40510-021-00367-3. |  |  |
| 4. | Sonone TP, Nawab A, Krishnaraj P, et al.; The Effects of Corticotomy and Piezocision in Orthodontic Canine Retraction: A Randomized Controlled Clinical Trial. J Pharm Bioallied Sci 2022;14(Suppl 1):S757-s764. doi: 10.4103/jpbs.jpbs_170_22. |  |  |
| 5. | Suryavanshi H, Das V, Deshmukh A, et al.; Comparison of rate of maxillary canine movement with or without modified corticotomy facilitated orthodontic treatment: A prospective clinical trial. APOS Trends in Orthodontics 2015;5. doi: 10.4103/2321-1407.159410. |  |  |
| 6. | Wang X, Mei M, Han G, et al.; Effectiveness of modified periodontally accelerated osteogenic orthodontics in skeletal class II malocclusion treated by a camouflage approach. Am J Transl Res 2022;14(2):979-989. |  |  |
| 7. | Addanki P, Gooty JR, Palaparthy R; Clinical and Radiographic Comparative Evaluation of Buccal and Palatal Corticotomy with Buccal Corticotomy in Periodontally Accelerated Osteogenic Orthodontics with Surgical Bur. Contemp Clin Dent 2017;8(2):321-326. doi: 10.4103/ccd.ccd_846_16. | Without a control group |  |
| 8. | Bahammam MA; Effectiveness of bovine-derived xenograft versus bioactive glass with periodontally accelerated osteogenic orthodontics in adults: a randomized, controlled clinical trial. BMC Oral Health 2016;16(1):126. doi: 10.1186/s12903-016-0321-x. |  |  |
| 9. | Brugnami F, Caiazzo A, Mehra P; Can corticotomy (with or without bone grafting) expand the limits of safe orthodontic therapy? J Oral Biol Craniofac Res 2018;8(1):1-6. doi: 10.1016/j.jobcr.2017.11.001. |  |  |
| 10. | Chen Z, Zhou H, Zhang K, et al.; The clinical efficacy of periodontally accelerated osteogenic orthodontics in patients with bone fenestration and dehiscence: a retrospective study. Head Face Med 2022;18(1):40. doi: 10.1186/s13005-022-00344-z. |  |  |
| 11. | Kabbur KJ, Kamaraj S, B R, et al.; An Assessment and Comparison of Root Resorption With Two Different Corticotomy Techniques During Anterior Teeth Retraction: A Split Mouth Prospective Clinical Study. Cureus 2023;15(1):e33431. doi: 10.7759/cureus.33431. |  |  |
| 12. | Kharkar V, Kotrashetti S; Transport dentoalveolar distraction osteogenesis-assisted rapid orthodontic canine retraction. Oral surgery, oral medicine, oral pathology, oral radiology, and endodontics 2010;109:687-93. doi: 10.1016/j.tripleo.2009.10.014. |  |  |
| 13. | Ma ZG, Yang C, Xie QY, et al.; A Novel Surgical Technique for Augmented Corticotomy-Assisted Orthodontics: Bone Grafting With Periosteum. J Oral Maxillofac Surg 2016;74(1):170-80. doi: 10.1016/j.joms.2015.06.147. |  |  |
| 14. | Panainte I, Nenovici DH, Mariș M, et al.; Morphologic Evaluation of Dentoalveolar Structures after Corticotomy-Assisted Orthodontic Treatment in Romanian Adult Patients. Medicina (Kaunas) 2022;58(4). doi: 10.3390/medicina58040468. |  |  |
| 15. | Sabban H; CORRELATION BETWEEN ROOT RESORPTION AND FAST ORTHODONTIC RETRACTION WITH CORTICOTOMY: A PILOT STUDY DONE BY USING CONE BEAM COMPUTED TOMOGRAPHY. Egyptian Dental Journal 2020. doi: 10.21608/edj.2020.29619.1124. |  |  |
| 16. | Shoreibah EA, Ibrahim SA, Attia MS, et al.; Clinical and radiographic evaluation of bone grafting in corticotomy-facilitated orthodontics in adults. J Int Acad Periodontol 2012;14(4):105-13. |  |  |
| 17. | Thind SK, Chatterjee A, Arshad F, et al.; A clinical comparative evaluation of periodontally accelerated osteogenic orthodontics with piezo and surgical bur: An interdisciplinary approach. J Indian Soc Periodontol 2018;22(4):328-333. doi: 10.4103/jisp.jisp_359_16. |  |  |
| 18. | Liu X, Fan B, Abdelrehem A, et al.; Membrane fixation for osseous graft stabilization in periodontally accelerated osteogenic orthodontics: a comparative study. BMC Oral Health 2020;20(1):22. doi: 10.1186/s12903-019-0964-5. |  |  |
