# Supplementary material for: Does invasive surgical acceleration of orthodontic tooth movement increase root resorption? A harms-focused systematic review
Source: PLoS One. 2026 Aug 19;21(8):e0356175. doi: 10.1371/journal.pone.0356175 (PMC13489456; doi:10.1371/journal.pone.0356175)
Supplement: Supplementary file 4 [file pone.0356175.s004.docx]

| **S4 Table.** Risk of bias of the included RCTs in this systematic review with supporting reasons | | | | | | | | | | | | |
| --- | --- | --- | --- | --- | --- | --- | --- | --- | --- | --- | --- | --- |
| **Study (Year/Country/Design)** | **Domain 1: Randomization process** | **Rationale (D1)** | **Domain 2: Deviations from intended interventions** | **Rationale (D2)** | **Domain 3: Missing outcome data** | **Rationale (D3)** | **Domain 4: Measurement of the outcome** | **Rationale (D4)** | **Domain 5: Selection of the reported result** | **Rationale (D5)** | **Overall judgment** |  |
| **Abbas et al**  **2016** | **Some concerns:** | Some concerns: Randomization sequences were generated using coin tosses. There was no information on the allocation sequence | **Low** | Low risk. Blinding of participants and people delivering the intervention cannot be performed. We judge that the outcome is not likely to be influenced by a lack of blinding | **Low** | Low risk: No dropouts were reported | **High risk** | High risk: No details of blinding the measurement stage (data collection) and the data analyzer | **Low** | Low risk: The protocol was not registered on ClinicalTrials.gov. But the reported outcomes in the results section seemed to be corresponding with the pre-defined outcomes aforesaid in the method section. | **High risk** |  |
| **Alam et al**  **2025** | **Some concerns** | Random allocation to ACAO vs control stated; CBCT assessors were blinded to group, but allocation concealment and sequence generation details are not reported. | **Low** | No evidence of protocol deviations; activation timing described (ACAO activated 1 week post-surgery; conventional timeline for control). | **Low** | No important loss to follow-up is reported; between-group comparisons are presented. | **Low** | CBCT at baseline and end, two independent examiners **blinded** to group—minimizes measurement bias. | **Some concerns** | No preregistered protocol or SAP is mentioned. | **Some concerns** |  |
| **Baiomy et al**  **2021** | **Some concerns** | Randomizer.org used to allocate the type of corticotomy (vertical vs modified). The opposite side served as **control**, i.e., side assignment to “surgery vs control” is not randomized → potential side-selection bias. | **Some concerns** | Within-mouth comparison (test vs control side) may introduce local RAP spillover; operators were not reported as blinded. Forces/anchorage standardized (150 g; miniscrews). | **Low** | 6-month follow-up with no material attrition reported. | Some concerns | “Root resorption parameters” are reported, but imaging modality/blinded assessors are not clearly specified (CBCT not explicitly stated). | **Some concerns** | No protocol registration reported; multiple outcomes with no pre-specified analysis plan. | **Some concerns** |  |
| **Raza et al**  **2021** | **Low** | Computer-generated random numbers by an independent person; **opaque sealed envelopes** used; side assignment revealed on surgery day. | **Low** | Uniform mechanics (150 g NiTi coils, anchor unit) and symmetric management; no deviations indicated. | **Low** | No missing data reported; complete paired comparisons. | **Low** | Root resorption measured on CBCT by an examiner **blinded to the side**. | **Some concerns** | No preregistration noted, though outcomes were pre-specified. | **Low** |  |
| **Sharma et al**  **2020** | **Some concerns** | Declared as randomized split-mouth, but sequence generation and concealment were not described. | **Low** | Standardized 150 g force; identical recall; no evidence of deviations. | **Low** | CONSORT flowchart shows 14 analyzed; no important attrition. | **Some concerns** | Root resorption assessed on IOPAR with Malmgren index; blinding reported for model-analyst/statistician but **not** explicitly for radiographic assessor. | **Some concerns** | No preregistration reported. | **Some concerns** |  |
| **Singh & Jayan**  **2019** | **Some concerns/High** | “Randomly distributed” into two groups is stated without details on sequence generation or concealment → potential allocation bias. | **Some concerns** | Constant mechanics described; no info on operator blinding. | **Low** | 40 participants (20/20) after applying criteria; no missing data signaled. | **High risk** | Root resorption via **Sharpe’s index** on radiographs; no statement that assessors were blinded to allocation → high risk of measurement bias. | **Some concerns** | No preregistered protocol referenced. | **High risk** |  |
