# Supplementary material for: Does invasive surgical acceleration of orthodontic tooth movement increase root resorption? A harms-focused systematic review
Source: PLoS One. 2026 Aug 19;21(8):e0356175. doi: 10.1371/journal.pone.0356175 (PMC13489456; doi:10.1371/journal.pone.0356175)
Supplement: Supplementary file 5 [file pone.0356175.s005.docx]

| **S5 Table. Risk of bias of the included Non-randomized Studies in this systematic review with supporting reasons** | | | | | | | | |
| --- | --- | --- | --- | --- | --- | --- | --- | --- |
| **Study (Country/Year/Design)** | **D1 Confounding** | **D2 Selection of participants** | **D3 Classification of interventions** | **D4 Deviations from intended interventions** | **D5 Missing data** | **D6 Measurement of outcomes** | **D7 Selection of the reported result** | **Overall** |
| **Ahn et al., 2016 (Korea, retrospective controlled)** — **Judgment** | **Serious** | **Moderate** | **Low** | **Low** | **Moderate** | **Moderate** | **Moderate** | **Serious** |
| **Justification** | Surgical allocation likely linked to severity/skeletal pattern/bone envelope; limited control → bias **favoring surgery** | Chart-based inclusion; baseline imbalance plausible | Presence/absence of surgery clearly documented | Routine care; no meaningful cross-overs | Potential incompleteness of some covariates/visits | Consistent method; assessor blinding unclear | No preregistration; possible selective emphasis | Overall, driven by confounding |
| **Ferguson et al., 2016 (UAE/USA, retrospective comparative)** — **Judgment** | **Serious** | **Moderate** | **Low** | **Low** | **Moderate** | **Moderate** | **Moderate** | **Serious** |
| **Justification** | PAOO choice tied to indication/preference (severity/bone quality/crowding); no robust adjustment → **favors PAOO** | Multicenter charts; likely baseline differences | Clear PAOO vs conventional definition | No protocol-deviant care | Possible incomplete records; no strong differential signal | Radiographic/clinical metrics consistent; blinding unclear | No preregistration; multiple outcomes/time points | Confounding predominates |
| **Hwang et al., 2017 (Korea, retrospective comparative) — Judgment** | **Serious** | **Moderate** | **Moderate** | **Moderate** | **Low** | **Moderate** | **Moderate** | **Serious** |
| **Justification** | Retrospective two-group study in an oral & maxillofacial surgery center; treatment allocation (ASO vs orthodontics only) was non-random and could be influenced by clinical/morphologic factors (age, sex, root shape, root–cortical relationship, treatment duration, etc.). The authors compared groups without multivariable adjustment (only t-tests and Mann–Whitney), and these very factors were discussed as determinants of resorption in the discussion. | Clear definition of groups: A = conventional orthodontics after extraction of maxillary first premolars; B = orthodontics + ASO with the same extractions. Misclassification is unlikely. No crossover reported. | Sample drawn from a single university oral surgery department (2007–2013), 28 patients (5 males/23 females; 19–29 years). This may introduce selection bias related to surgical access and clinical preferences. | Retrospective design; no detailed information on patient adherence or unreported co-interventions. | Two time-points with CBCT (T0/T1); no missing outcome data reported | Objective CBCT tooth-length metrics; assessor blinding not stated. | No preregistered protocol/analysis plan mentioned; multiple outcomes/time points reported | Confounding predominates |
| **Ma et al., 2023 (China, prospective non-randomized controlled)** — **Judgment** | **Serious** | **Moderate** | **Low** | **Low** | **Low** | **Moderate** | **Moderate** | **Serious** |
| **Justification** | Non-random allocation with likely baseline imbalances (age/severity/root length/goal); no robust matching/weighting → may **favor surgery** | Clinical pre-selection; no immortal-time signals | Clear definition/timing of augmented corticotomy vs conventional | Uniform mechanics; no cross-overs | No material attrition | CBCT objective: assessor blinding not explicit | No preregistered protocol/SAP; multiple teeth/outcomes | Overall, driven by confounding |
| **Wang et al., 2013 (China, prospective non-randomized)** — **Judgment** | **Serious** | **Moderate** | **Low** | **Low** | **Moderate** | **Moderate** | **Moderate** | **Serious** |
| **Justification** | Non-random allocation likely yields baseline imbalances (age/severity/root length, etc.) with no stated comprehensive adjustment → potential bias **favoring one group** | Clinical pre-selection before intervention may link to prognosis | Group definitions/timing are typically clear in comparative designs | No indications of cross-overs; routine care | Full text not available here → prudent assumption of some missingness | Outcome method not available to us; assume radiographic; **assessor blinding unclear** | No info on preregistration; multiple teeth/timepoints → plausible selective reporting | Provisional overall rating driven by confounding, pending full-text review |
