# Supplementary material for: Does invasive surgical acceleration of orthodontic tooth movement increase root resorption? A harms-focused systematic review
Source: PLoS One. 2026 Aug 19;21(8):e0356175. doi: 10.1371/journal.pone.0356175 (PMC13489456; doi:10.1371/journal.pone.0356175)
Supplement: Supplementary file 6 [file pone.0356175.s006.docx]

| **S6 Supplementary Table.** McMaster Quality Assessment Scale of root resorption of the included **RCTs** and **Non-randomized Studies** in this systematic review, with supporting reasons | | | | | | | | | | | |
| --- | --- | --- | --- | --- | --- | --- | --- | --- | --- | --- | --- |
|  | **1** | **2** | **3** | **4** | **5** | **6** | **7** | **8** | **9** | **10** | **11** |
| **Abbas et al**  **2016** | Root resorption has not been pre-defined and studied as a **secondary** objective | The **mm** unit was used to determine the root resorption intensity | Appliance and intervention characteristics have been **explained** | It has **not** been specified who collected the root resorption data | There **has not been** training, background, or reliability study of who ascertained the root resorption | The phrase ‘'before and after retraction’’ has been mentioned and the **duration** of the retraction has been determined | No clear standard scale has been used for root resorption values | RCT study. There is **no** a diagram showing the flow of participants. And there is **no** indicated that all patients completed the study phases | There is mentioned the total number of teeth with root resorption | **Apical** root resorption has been studied | The type of analysis undertaken for root resorption data has **not** been specified. |
| **Ahn et al**  **2016** | Root resorption has been pre-defined and studied as a **secondary** objective | The **mm** unit was used to determine the root resorption intensity | Appliance and intervention characteristics have been **explained** | It has been specified who collected the root resorption data | **Reliability has been studied** (without mentioning the training or background of who ascertained the root resorption). | The phrase ‘Baseline & post-decompensation’’ has been mentioned and the **duration** has been determined | Standard scale has been used for root resorption values | Retrospective study—a participant flow diagram is typically not expected. No flow diagram is provided, and withdrawals are not reported/applicable since cohorts were identified from completed records. | There is mentioned the total number of teeth with root resorption | **Apical** root resorption has been studied | The type of analysis undertaken for root resorption data has been specified.  **(t-test, ANOVA)** |
| **Alam et al**  **2021** | Root resorption has not been pre-defined and studied as a **primary** objective | The **mm** unit was used to determine the root resorption intensity Without specifying the measurement method | Appliance and intervention characteristics have been **explained** | The phrase “**two independent examiners blinded**” was mentioned without mentioning his name | There **has not been** training, background, or reliability study of who ascertained the root resorption | The phrase ‘Baseline & post-treatment’’ has been mentioned and the **duration** has been determined | The paper does not provide a detailed RR measurement protocol (no landmarks/tool/settings/unit), so another researcher could not reproduce it from the text. | RCT design—no participant flow diagram is shown, and there is no statement that all patients completed all study phases. | There is **no** mentioned the total number of teeth with root resorption | **Apical** root resorption has been studied | The type of analysis undertaken for root resorption data has been specified.  **(t-test)** |
| **Baiomy et al**  **2021** | Root resorption has been pre-defined and studied as a **secondary** objective | The **mm** unit was used to determine the root resorption intensity Without specifying the measurement method | Appliance and intervention characteristics have been **explained** | It has **not** been specified who collected the root resorption data | There **has not been** training, background, or reliability study of who ascertained the root resorption | The phrase ‘Baseline & after retraction’' has been mentioned and the **duration** has been determined | The paper does not provide a detailed RR measurement protocol (no landmarks/tool/settings/unit), so another researcher could not reproduce it from the text. | Split-mouth RCT—no flow diagram is presented, and there is no explicit indication that all participants completed every phase. | There is mentioned the total number of teeth with root resorption | **Apical** root resorption has been studied | The type of analysis undertaken for root resorption data has been specified.  **(Paired/unpaired t)** |
| **Ferguson et al**  **2016** | Root resorption has been pre-defined and studied as a **primary** objective | The **mm** unit was used to determine the root resorption intensity | Appliance and intervention characteristics have been **explained** | It has **not** been specified who collected the root resorption data | **Reliability has been studied** (without mentioning the training or background of who ascertained the root resorption). | The phrase ‘'before and after treatment’’ has been mentioned and the **duration** has been determined | Standard scale has been used for root resorption values | Retrospective comparative—a flow diagram is generally not required; none is provided, and no withdrawals are reported because cohorts derive from completed charts. | There is mentioned the total number of teeth with root resorption | **Apical** root resorption has been studied | The type of analysis undertaken for root resorption data has been specified.  (**t-test**) |
| **Hwang et al**  **2017** | Root resorption has been pre-defined and studied as a **primary** objective | The **mm** unit was used to determine the root resorption intensity | Appliance and intervention characteristics have been **explained** | It has **not** been specified who collected the root resorption data | There **has not been** training, background, or reliability study of who ascertained the root resorption | The phrase ‘’Two points: pre-treatment (T0) and end of active treatment (T1)’’ has been mentioned and the **duration** has been determined | Standard scale has been used for root resorption values | Non-randomized CCT—no participant flow diagram is provided, and there is no explicit statement that all participants completed all phases/follow-up. | There is mentioned the total number of teeth with root resorption | **Apical** root resorption has been studied | The type of analysis undertaken for root resorption data has been specified.  (**Mann–Whitney U**) |
| **Ma et al**  **2023** | Root resorption has been pre-defined and studied as a **secondary** objective | The **mm** unit was used to determine the root resorption intensity | Appliance and intervention characteristics have been **explained** | It has **not** been specified who collected the root resorption data | There **has not been** training, background, or reliability study of who ascertained the root resorption | The phrase ‘pre vs post presurgical phase’’ has been mentioned and the **duration** has been determined | Standard scale has been used for root resorption values | Non-randomized CCT—no participant flow diagram is provided, and there is no explicit statement that all participants completed all phases/follow-up. | There is mentioned the total number of teeth with root resorption | **Apical** root resorption has been studied | The type of analysis undertaken for root resorption data has been specified.  (**t-test**) |
| **Raza et al**  **2021** | Root resorption has been pre-defined and studied as a **secondary** objective | The **mm** unit was used to determine the root resorption intensity | Appliance and intervention characteristics have been **explained** | The phrase “**one examiner blinded**” was mentioned without mentioning his name | There **has not been** training, background, or reliability study of who ascertained the root resorption | The phrase ‘'before and after retraction’’ has been mentioned and the **duration** has been determined | Standard scale has been used for root resorption values | RCT design—no flow diagram is included, and there is no explicit confirmation that all participants completed all phases. | There is mentioned the total number of teeth with root resorption | **Apical** root resorption has been studied | The type of analysis undertaken for root resorption data has been specified.  (**t-test)** |
| **Sharma et al**  **2020** | Root resorption has been pre-defined and studied as a **secondary** objective | The **Malmgren scoring index** unit was used to determine the root resorption intensity | Appliance and intervention characteristics have been **explained** | The phrase “**one examiner**” was mentioned without mentioning his name | **Reliability has been studied** (without mentioning the training or background of who ascertained the root resorption). | The phrase ‘'before and after retraction’’ has been mentioned and the **duration** has been determined | Standard scale has been used for root resorption values | RCT design—a CONSORT flow diagram is included, and attrition is reported (17 enrolled, 14 analyzed). | There is mentioned the total number of teeth with root resorption | **Changes in Malmgren scoring index** have been studied | The type of analysis undertaken for root resorption data has been specified.  (**t-test, Chi²)** |
| **Singh & Jayan.**  **2019** | Root resorption has not been pre-defined and studied as a **secondary** objective | The **Sharpe index** unit was used to determine the root resorption intensity | Appliance and intervention characteristics have been **explained** | It has **not** been specified who collected the root resorption data | There **has not been** training, background, or reliability study of who ascertained the root resorption | The phrase ‘Baseline & post-treatment’’ has been mentioned and the **duration** has been determined | Standard scale has been used for root resorption values | RCT design—no flow diagram is included, and there is no explicit confirmation that all participants completed all phases. | There is mentioned the total number of teeth with root resorption | **Changes in Sharpe index** have been studied | The type of analysis undertaken for root resorption data has been specified.  **(Mann-Whitney, Wilcoxon)** |
| **Wang et al**  **2013** | Root resorption has been pre-defined and studied as a **secondary** objective | The **mm** unit was used to determine the root resorption intensity | Appliance and intervention characteristics have been **explained** | The phrase “**one examiner**” was mentioned without mentioning his name | **Reliability has been studied** (without mentioning the training or background of who ascertained the root resorption). | The phrase ‘‘pre vs post presurgical phase’’ has been mentioned and the duration has been determined | Standard scale has been used for root resorption values | Non-randomized comparative design—no flow diagram is included (not typically required), and explicit per-group completion/attrition is not reported. | There is mentioned the total number of teeth with root resorption | **Apical** root resorption has been studied | The type of analysis undertaken for root resorption data has been specified.  (**t-tests**) |
